# Multiscale Dissection of Spatial Heterogeneity by Integrating Multi‐Slice Spatial and Single‐Cell Transcriptomics

**DOI:** 10.1002/advs.202413124

**Published:** 2025-02-25

**Authors:** Yuqi Chen, Caiwei Zhen, Yuanyuan Mo, Juan Liu, Lihua Zhang

**Affiliations:** ^1^ School of Computer Science Wuhan University Wuchang District Wuhan Hubei 430072 China

**Keywords:** cell type deconvolution, multiscale structure, spatial transcriptomics, scRNA‐seq, spatial domain

## Abstract

The spatial structure of cells is highly organized at multiscale levels from global spatial domains to local cell type heterogeneity. Existing methods for analyzing spatially resolved transcriptomics (SRT) are separately designed for either domain alignment across multiple slices or deconvoluting cell type compositions within a single slice. To this end, a novel deep learning method, SMILE, is proposed which combines graph contrastive autoencoder and multilayer perceptron with local constraints to learn multiscale and informative spot representations. By comparing SMILE with the state‐of‐the‐art methods on simulation and real datasets, the superior performance of SMILE is demonstrated on spatial alignment, domain identification, and cell type deconvolution. The results show SMILE's capability not only in simultaneously dissecting spatial variations at different scales but also in unraveling altered cellular microenvironments in diseased conditions. Moreover, SMILE can utilize prior domain annotation information of one slice to further enhance the performance.

## Graphical abstract

Integrating multiple spatial transcriptomics datasets can provide a more comprehensive understanding of tissue structure and cellular heterogeneity. However, batch effects between different slices as well as between spatial transcriptomics and single‐cell reference data bring a great challenge for the integrative analysis. SMILE is a deep learning‐based method, which can not only align multiple slices but also reveal cellular composition.

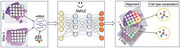

## 1 Introduction

The functionality of cells is intricately linked to their spatial organizations, and the advent of spatial transcriptomics (SRT) technologies has furnished robust tools for exploring the intricate spatial distribution of cells. Biological systems are dynamic at multiple scales, ranging from molecular and cellular to tissue levels. At the cellular level, single‐cell RNA‐sequencing (scRNA‐seq) provides a direct window into the molecular makeup of individual cells, enabling the detailed characterization of cell type heterogeneity. At the tissue level, advances in SRT help to map cells and their molecular states onto tissues and organize cells into spatially functional domains, improving our understanding of key physiological processes. SRT technologies, particularly sequencing‐based technologies like 10X Visium,^[^ ^1^ ^]^ Slide‐seq,^[^ ^2^ ^]^ HDST,^[^ ^3^ ^]^ and Stereo‐seq,^[^ ^4^ ^]^ not only enable the capture of gene expression profiles of tens of thousands of genes but also permit precise spatial localization. However, understanding cellular heterogeneity and tissue organization from such SRT data remains a challenges due to the sparse characteristics of the data.^[^ ^5^ ^]^ The integration of tissue slices derived from diverse sources such as different anatomical sites, biological replicates and experimental conditions facilitates the exploration of spatial heterogeneity^[^ ^6^ ^]^ yet remains a great challenge due to the batch effects.^[^ ^5^ ^]^

To better align spatial domains from multiple SRT data, several deep learning methods have been developed to integrate tissue slices from different sources, such as STAligner,^[^ ^7^ ^]^ SLAT,^[^ ^8^ ^]^ GraphST,^[^ ^9^ ^]^ and IRIS.^[^ ^10^ ^]^ STAligner utilizes a triplet construction to guide the model in effectively mitigating batch effects of low‐dimensional representations derived from a graph attention autoencoder. SLAT uses a graph‐based adversarial matching strategy, facilitating the alignment of heterogeneous slices across distinct technologies and modalities. By leveraging graph self‐supervised contrastive learning and a feature smoothing strategy within both intra‐ and inter‐sample neighbors, GraphST can align multiple slices to identify spatial domains, and is also applicable for deconvoluting spots through the integration of spatial and single cell transcriptomic data. However, these two tasks are separately designed in GraphST and the deconvoluted cellular compositions are not comparable across different slices. Different from these methods, IRIS uses scRNA‐seq data for reference‐informed detection of spatial domains, leading to improved accuracy compared to those based only on SRT data. However, it may not be applicable for accurately deconvoluting spatial transcriptomic data from multiple slices. Moreover, these methods are unsupervised, thereby disregarding the potential advantage of utilizing labeled data from certain slices in scenarios where biologists can manually annotate the spatial regions of one of the multiple slices.

The aforementioned methods provide insights into the spatially organized domains on tissues but lose the ability to reveal the cellular heterogeneity within spatial domains. Several computational methods have been proposed to estimate cell type compositions within each spot from one SRT data by taking scRNA‐seq data with known cell type labels as a reference. For example, STEP,^[^ ^11^ ^]^ RCTD,^[^ ^12^ ^]^ SPOTlight,^[^ ^13^ ^]^ cell2location,^[^ ^14^ ^]^ CARD^[^ ^15^ ^]^ and SpaOTsc^[^ ^16^ ^]^ have been developed. CARD and Cell2location are two top‐performing methods based on the previous benchmarking study.^[^ ^17^ ^]^ However, they are not applicable for accurately deconvoluting spatial transcriptomic data from multiple slices as the existence of batch effects between different slices and batch effects between spatial and single cell transcriptomic data,^[^ ^5^ ^]^ which hampers the efforts for revealing differential cellular compositions and microenvironments across different conditions like normal versus disease.

To address these limitations, we propose a deep learning method SMILE to simultaneously **i** ntegrate SRT from mu **l** tiple slices and scRNA‐seq reference data. SMILE can characterize the spatial heterogeneity of cells organized at multiscale levels from global spatial domains to local cell type heterogeneity. Through applications to both simulated and three published datasets and comparisons with existing SRT alignment and deconvolution methods, SMILE is found to be an efficient approach to reveal spatial heterogeneity at both domain and cellular levels. We also present a semi‐supervised version of SMILE, named SMILE‐simi, and show the advantage of utilizing prior domain annotation information of one slice to enhance the performance when aligning multiple slices of SRT data. Moreover, we demonstrate the capability of SMILE in revealing altered cellular composition and microenvironment signaling in the disease condition by applying it to spatial transcriptomics data from healthy and psoriatic skin.

## 2 Results

### 2.1 Overview of SMILE

**Figure 1:**
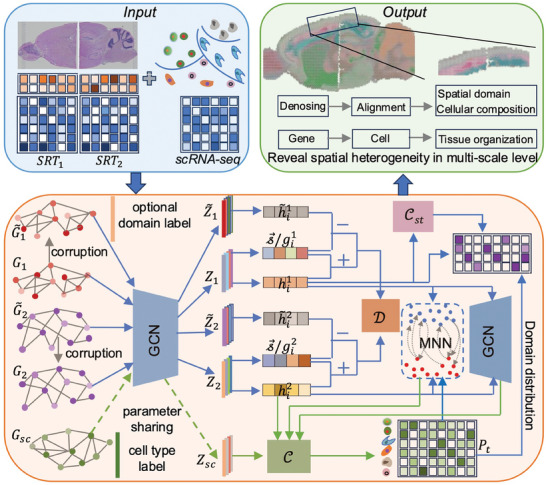
Workflow of SMILE. The input data for SMILE are the gene expression profiles of spots, spot coordinates and scRNA‐seq reference data. The core algorithm of SMILE includes two key modules, one is for SRT data modeling indicated by blue lines and the other is for scRNA‐seq data modeling marked by green lines. Dashed green lines represent parameter sharing strategy. For each SRT data, the graph *G_i_* is built based on spot coordinates, the corrupted graph ${\tilde{G}}_{i}$ is constructed by randomly permuting nodes’ features, and the low‐dimensional embedding *Z_i_* of each graph is obtained by using a two‐layer GCN module. $C_{st}$ is a multi‐layer perceptron classifier for SRT data if the domain label is available. D is a bilinear discriminator and $C_{sc}$ is a multi‐layer perceptron classifier for cell type representations of spots. *P_t_* is the cell type representation of one slice, providing the cell type proportions of each spot in this slice. MNN indicates anchor pairs of different slices identified by mutual nearest neighbors based on spot embeddings or cell type representations. The outputs of SMILE are denoised SRT data, aligned embeddings of different slices, identified spatial domains and estimated cell type compositions of each spot.

SMILE takes SRT data of multiple slices and scRNA‐seq reference data as inputs (**Figure** **1**). SMILE builds a spatial graph among spots of each slice using an alpha‐complex‐based method based on the spot coordinates (Experimental Section), and further constructs a corrupted graph by randomly permuting nodes’ features for each spatial graph (Experimental Section). Then SMILE learns low dimensional embeddings of spots by utilizing a two‐layer graph convolutional network (GCN) on each graph and employing contrastive learning between the spatial graph and its corrupted graph. The latent embedding of each slice is used to reconstruct the corresponding gene expression profile via a GCN decoder, leading to denoised SRT data.

To make the embeddings of spatial transcriptomics data and scRNA‐seq data comparable, we first learned the parameters of the graph convolutional neural network based on spatial transcriptomics data, and then extracted the embedding of scRNA‐seq data using the graph convolutional neural network with the learned parameters. Specially, the graph of scRNA‐seq data was built using the shared nearest neighbor (SNN) graph of single cells. SMILE then trains a classifier on the learned low dimensional embedding of single‐cell data. To transfer this classifier to the SRT data, SMILE introduces a cell type representation module that gives the cell type proportions of each spot to constrain this classifier. SMILE identifies spatial domains by applying the Gaussian finite mixture clustering (mclust) method on the cell type representations and treats this representation as the clustering prior of spots. To align similar spots coming from different slices, SMILE identifies anchors by building a mutual nearest neighbor (MNN) graph on the latent embeddings or cell type representations of spots, and minimizes the difference in the distribution of the latent embeddings of these anchors. Finally, SMILE outputs denoised SRT data, aligned low dimensional embeddings, spatial domains, and cell type representation of each spot.

SMILE is also applicable to the scenario when the spatial domain labels of at least one slice are available, which is termed as a SMILE‐semi method. Compared to the SMILE method, SMILE‐semi additionally employs a multi‐layer perceptron classifier for the slice with known domain labels, and then predicts the spatial domain labels for other slices by using this trained classifier, leading to better performance in a semi‐supervised manner. The SMILE‐semi method is particularly useful when biologists are able to manually annotate the spatial regions of one of the multiple slices.

### 2.2 SMILE Outperforms Other State‐of‐the‐Art Methods on Simulated Datasets

**Figure 2:**
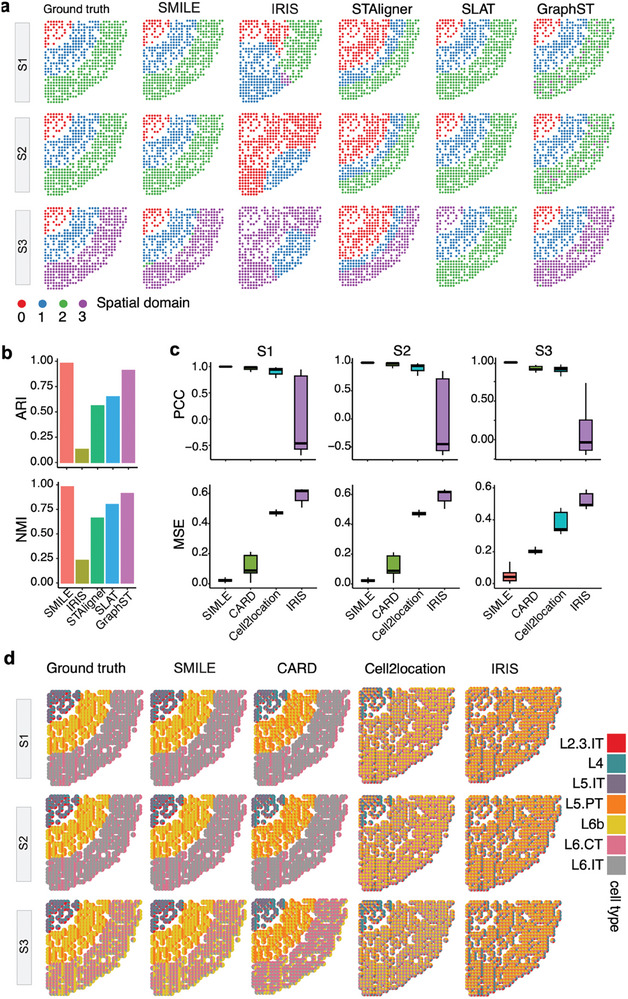
Comparison of SMILE against existing methods on simulation data. a) Spatial visualization of the domains of the ground truth and those identified by SMILE, IRIS, STAligner, SLAT, and GraphST. b) Evaluation of domain identification accuracy using ARI and NMI metrics. c) Comparison of deconvolution performance among different methods using two metrics PCC and MSE. d) Proportions of deconvolved cell types from ground truth, SMILE, CARD, Cell2location, and IRIS, respectively. Each spot is represented by a pie chart of cell‐type proportions.

To evaluate SMILE against other spatial domain detection and cell type deconvolution methods, we simulated SRT based on real scRNA‐seq data of mouse brain (Methods, Supporting Information), where the ground‐truth spatial domains and cell type compositions were available. To recapitulate the heterogenous properties of spatial transcriptomic data, we generated three slices of SRT data (i.e., Figures S1–S3, Supporting Information). As shown in the first column of **Figure** **2**a, each slice has three domains: S1 and S2 have similar cell type compositions, while one domain of S3 has different cell type compositions. First, we compared SMILE with the other four spatial transcriptomic data integration methods including IRIS, STAligner, SLAT, and GraphST. Both SMILE and GraphST successfully identified the spatial domains, except that GraphST assigned a few spots to incorrect domains (Figure 2a). IRIS failed to reveal these spatial domains, and STAligner exhibited poor performance in capturing the borders of nearby domains. SLAT exhibited good performance for slices S1 and S2, but failed to identify the specific domain of slice S3. Next, we used the Adjusted Rand Index (ARI) and normalized mutual information (NMI) to quantitively evaluate the performance of identifying spatial domains. As expected, SMILE has the largest ARI and NMI values compared to all other methods, and GraphST has slightly lower values than SMILE (Figure 2b).

Moreover, we evaluated the performance of SMILE in estimating the cell type proportions within each spot by comparing it against a recently proposed method IRIS and another two widely used spot deconvolution methods including CARD and Cell2location (Figure 2d). Visually, SMILE and CARD were able to reveal different cell type proportions across all spatial domains, but Cell2location and IRIS failed to do it. To quantitatively compare the performance of different methods, we calculated the Pearson correlation coefficients (PCC) and mean square errors (MSE) between the estimated cell type proportions and the ground truth. SMILE achieved the highest PCC values of 1 and the lowest MSE values approaching zero for all three slices, and CARD exhibited slightly worse performance compared to SMILE in terms of these metrics (Figure 2c). IRIS incorrectly estimated the cell type proportions, with the median PCC values of −0.5 for slices S1 and S2, and 0 for slice S3 Taken together, SMILE exhibited superior performance in simultaneously identifying the spatial domains and estimating the cell type proportions within spots, allowing the dissection of spatial heterogeneity at multiple scales compared to existing SRT analysis methods.

### 2.3 Domain Annotation Information Enhances Spatial Alignment on Spatial Data of Human Dorsolateral Prefrontal Cortex

To further benchmark SMILE against existing methods on real datasets, we applied SMILE to the human dorsolateral prefrontal cortex (DLPFC) 10x Visium datasets from different slices, where each slice was manually annotated with six DLPFC layers and white matter (WM) based on the morphological features and gene markers in the previous study.^[^ ^18^ ^]^ We also obtained the corresponding 10x Chromium scRNA‐seq data from post‐mortem brain tissue, with 28 annotated cell types as a reference.^[^ ^19^ ^]^ We treated the domain annotations from the previous study as the ground truth and compared SMILE against the ground truth, the non‐spatial integration method Scanpy,^[^ ^20^ ^]^ and four recently developed alignment methods: STAligner, SLAT, GraphST, and IRIS. In addition, we also evaluated the semi‐supervised method SMILE_semi by taking advantage of the domain annotation information from one slice.

**Figure 3:**
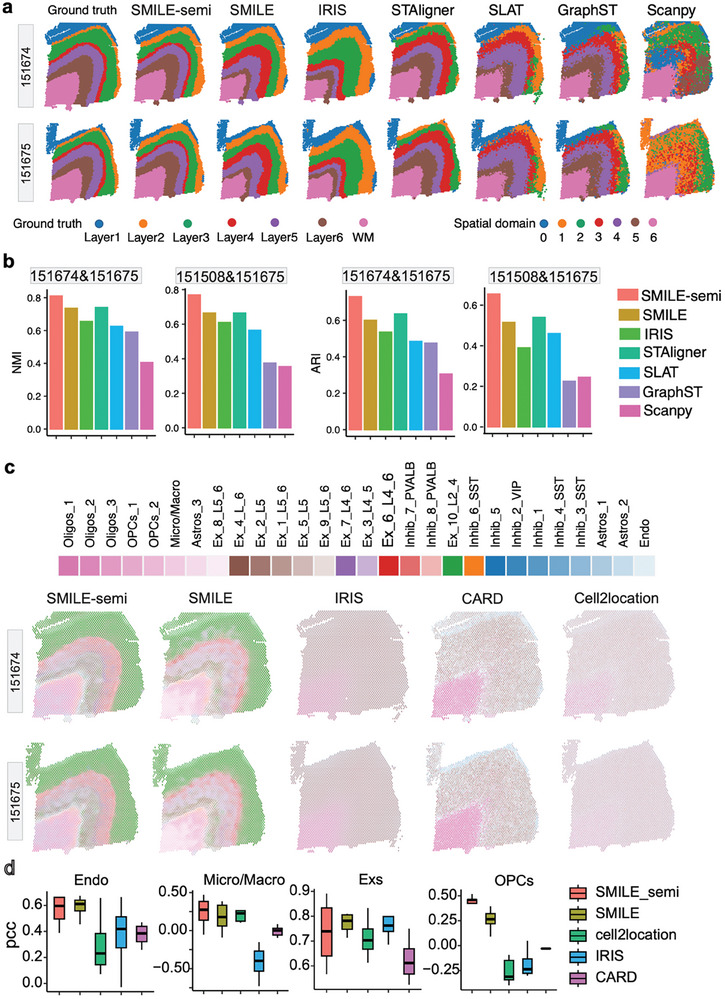
Evaluating performance of SMILE_semi and SMILE against existing methods on DLPFC datasets. a) The ground truth and spatial domains identified by seven methods on the slices of two DLPFC samples (151674 and 151675) from the same donors are shown. The manual annotations of cortical layers and white matter from the previous study are considered as ground truth. b) Barplots showing the accuracy of the identified spatial domains in terms of NMI and ARI scores. c) Mapping the deconvoluted cell type proportions from five methods onto tissues. The spatial scatter pie plot displays cell type proportions within spots. d) Boxplots showing Pearson correlation coefficients between the cell type proportions and the expression of marker genes for each major cell type.

We first evaluated the performance of all methods on the tissue slices coming from the same donor, which had similar tissue structures. Here, we took slices 151674 and 151675 as an example, and utilized the annotation information of slice 151674 to run SMILE_semi. Encouragingly, SMILE_semi was able to accurately recapitulate the known layered structures of the prefrontal cortex, and showed better performance compared to SMILE (**Figure** **3**a; Figure S1a,b, Supporting Information). SMILE‐semi accurately identified the rare layer 4, while all other methods had difficulty detecting this rare domain. Both SMILE and STAligner were able to roughly identify the different layers with clear layer boundaries, and predominantly outperformed the other four methods, including SLAT, GraphST, IRIS, and Scanpy. Scanpy failed to detect spatial layers, suggesting the importance of incorporating spatial information, and IRIS incorrectly assigned spots into spatial domains, suggesting the possible weakness of linear approaches in representing the complex tissue used in IRIS. Moreover, we quantitatively evaluated the spatial domain detection accuracy by utilizing two metrics ARI and NMI. Consistent with the visual examination, SMILE‐semi accurately captured the layer boundaries and had the largest ARI and NMI values compared to other methods. SMILE exhibited comparable ARI and NMI values compared to STAligner, but higher values than other methods, suggesting the superior performance of SMILE‐semi and SMILE in detecting spatial domains. Of note, the number of identified spatial domains may affect the ARI and NMI values. We found that when SMILE produced six spatial domains, the ARI improved from 0.6 to 0.655 and NMI improved from 0.735 to 0.755, which were higher than those of STAligner (ARI: 0.635, NMI: 0.74, Figure 2b; Figure S1d, Supporting Information).

We next applied SMILE to the tissue slices coming from different donors, which had large batch effects and brought additional challenges for domain detection (Figures S2 and S3, Supporting Information). We took slices 151508 and 151675 as an example and utilized the annotation information of slice 151508 to run SMILE_semi. Both SMILE_semi and SMILE successfully removed batch effects and separated distinct layers, as shown in the Uniform Manifold Approximation and Projection (UMAP) space^[^ ^21^ ^]^ (Figure S2a, Supporting Information). The classifier in the SMILE_semi module could accurately predict layers for slice 151675, including the rare layer 2 and layer 4 (Figure S2b, Supporting Information). Then we used the ARI and NMI metrics to quantitatively evaluate the domain identification performance. SMILE_semi achieved the highest ARI and NMI scores among all methods. STAligner and SMILE had higher NMI and ARI values than other methods (Figure 3b). GraphST significantly reduced its performance compared to the scenario of integrating slices from the same donors. In addition, only SMILE and SLAT clearly capture the WM layer of slice 151508, while other methods cannot detect the WM layer. Further examination showed that SLAT cannot well distinguish layers 4–6 (Figure S3a, Supporting Information). These results indicated the ability of SMILE_semi and SMILE to integrate different SRT datasets with large batch effects.

### 2.4 SMILE Unravels the Spatially Informed Cellular Heterogeneity of Human Dorsolateral Prefrontal Cortex Layers

By leveraging scRNA‐seq reference data, SMILE could investigate the spatial domains at a cellular level, leading to the characterization of cell type compositions within each spot and domain. We evaluated SMILE by comparing it with CARD, Cell2location and IRIS, where each method outputs the composition of cell types within each spot. By representing the cell type proportions of each spot using a pie chart, we found that SMILE_semi and SMILE succeeded in predicting regularly distributed cell types on the tissue. However, CARD and IRIS only predicted the enrichment of Oligos in the white matter and failed to capture the spatial patterns of other cell types. Cell2location produced no obvious patterns of any cell type (Figure 3c). Due to the lack of ground truth in cell type compositions, we identified the top 20 marker genes of each major cell type based on scRNA‐seq reference data, and then computed Pearson correlation coefficients between the marker genes’ expression and the normalized proportions of each major cell type, where the cell type proportions across all spots were normalized to be 1. Expectedly, SMILE_semi and SMILE had larger PCC values than other methods for each cell type (Figure 3d; Figure S4, Supporting Information).

**Figure 4:**
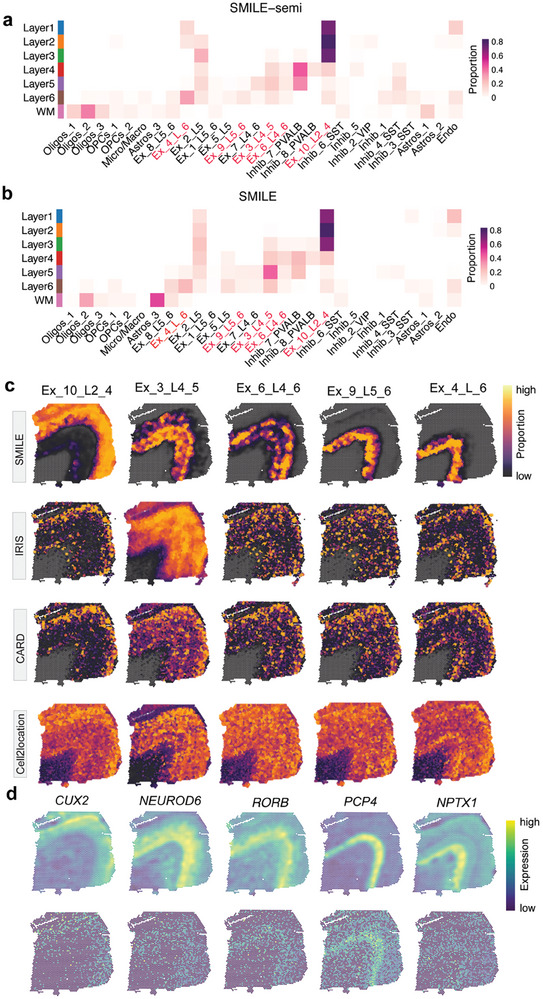
SMILE unravels the spatially informed cellular heterogeneity of human dorsolateral prefrontal cortex layers. a) and b) Heatmap plots displaying the estimated mean cell type proportions by SMILE_semi and SMILE in each layer, respectively. c) A spatial scatter plot displaying the spatial distribution of the cell type proportions of indicated cell types across spatial locations, as inferred by SMILE_semi, SMILE, IRIS, CARD, and Cell2location, respectively. d) Spatial feature plots showing the expression of representative marker genes including CUX2, NEUROD6, RORB, PCP4, and NPTX1, respectively. The denoised (upper panel) and original (lower panel) gene expressions of these marker genes are shown for comparison.

Next, we systematically and quantitatively investigated the cell type's spatial distribution across different layers. We found that Oligio_1, Oligio_2, and Oligo_3 were distributed in the white matter, Ex_4_L_6 was enriched in layer 6 (**Figure** **4**a,b), which were consistent with previously observed enrichment of these cell types in the corresponding cortical layers.^[^ ^22^ ^]^ Then we inspected the spatial distribution of each cell type identified by SMILE and other deconvolution methods on slices 151674 and 151508 (Figures S5–S13, Supporting Information). Here, we took Ex_10_L2_4, Ex_3_L4_5, Ex_6_L4_6, Ex_9_L5_6, and Ex_4_L_6 as examples (Figure 4c). We found that SMILE could distinguish the progressive layering of the cortical regions, but IRIS, CARD, and Cell2location predicted that these cortex cell types were much more scattered and did not correspond to their expected positions in the layers. Next, we performed differential expression (DE) analysis to characterize the cellular landscape of tissue domains. For example, CUX2 is an identified DE gene in Ex_10_L2_4, which is primarily expressed in nervous tissues and selectively regulates the number of upper layer cortical neurons.^[^ ^23^ ^]^ NEUROD6 is a key regulator of fasciculation and targeted axogenesis in the mouse neocortex,^[^ ^24^ ^]^ which is located in Ex_3_L4_5. RORB is an identified DE gene in Ex_3_L4_6, which is a marker of layer 4 (L4) neurons. PCP4 is an identified DE gene in Ex_9_L5_6 and PCP4's expression is generally enriched in layers 5 and 6.^[^ ^25^ ^]^ NPTX1 is a member of the neuronal pentraxin gene family, which is an identified DE gene of Ex_4_L_6. Moreover, SMILE could clearly denoise and enhance the expression patterns of these markers (Figure 4d). These results indicated the ability of SMILE in precise cell type deconvolution and characterizing biologically meaningful organization of cell types on tissues. Taken together, the comprehensive analyses of DLPFC demonstrated 1) the superior performance of SMILE in both spatial domain identification and cell type deconvolution, highlighting the SMILE as a unique method to simultaneously dissect spatial variations at different scales compared to existing methods; and 2) the advantage of utilizing prior domain annotation information of one slice to enhance both analysis tasks.

### 2.5 SMILE Accurately Reveals Spatially Continuous Heterogeneity by Jointly Analyzing Anterior and Posterior Sections of Mouse Brain

**Figure 5:**
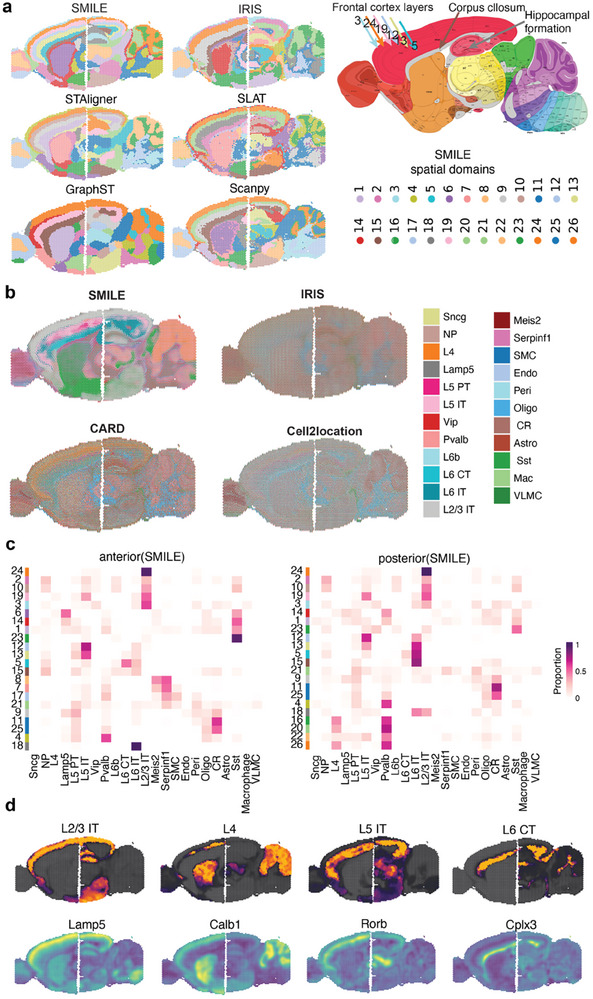
SMILE identifies spatially patterned domains and cell states across adjacent slices of mouse brain. a) Joint analysis of two consecutive tissue sections from the mouse anterior and posterior brain by SMILE, IRIS, SLAT, STAligner, GraphST and Scanpy, respectively. Allen Brain Institute reference atlas diagram of the mouse cortex and the subdomain manual annotation (right). b) Results of mapping spatial data to single‐cell data. The spatial scatter pie plot displays the well‐structured cluster composition. c) Heatmap plot displaying the estimated mean cell type proportion for each cell type in each spatial domain detected by SMILE for the anterior and posterior slice, respectively. The color scale was normalized to the 0–1 range. d) A spatial scatter plot displaying the spatial distribution of the SMILE estimated cell type proportion for L2/3 IT, L4, L5 IT, and L6 CT across spatial locations. The bottom shows the denoised spatial expression of marker gene Lamp5, Calb1, Rorb, and Cplx3, respectively.

To evaluate SMILE's ability in concatenating contiguous regions, we applied SMILE to the spatial transcriptomic datasets of mouse anterior and posterior brain generated by the 10x Visium platform. The anterior and posterior sections are adjacent in the brain. Using spatial transcriptomic data of these two slices, SMILE can achieve a complete depiction of the whole sagittal mouse brain in an unsupervised clustering manner (**Figure** **5**a). We used the Allen Brain Atlas reference to annotate anatomical regions such as the frontal cortex layers, corpus callosum and hippocampal formation. Most methods except for IRIS could identify contiguous cortex layers across anterior and posterior sections. Notably, SMILE was also able to reveal continuous corpus callosum and hippocampal formation structures, while other methods failed, suggesting a better ability of SMILE to decipher the precise functional domains.

snRNA‐seq using the 10X Genomics Chromium platform on dorsolateral prefrontal cortex tissue is available from the previous study.^[^ ^26^ ^]^ We further used SMILE to explore the sagittal mouse brain at the cellular level by integrating the spatial transcriptomic data and snRNA‐seq data. SMILE successfully mapped the relevant cell types in the scRNA‐seq data onto the spatial locations (Figure 5b). Compared with IRIS, CARD,^[^ ^15^ ^]^ and Cell2location,^[^ ^14^ ^]^ SMILE's mapping was clearer with sharper boundaries. On the contrary, most of the cell types exhibited no obvious patterns in the deconvolution results of IRIS, CARD, and Cell2location's mappings. To quantify the relationship between the identified spatial domains and the deconvoluted cell types, we computed the mean proportion of each cell type among each spatial domain (Figure 5c). Astro cells were enriched in domain 3, which represented the outer layer of the cortex. L2/3 IT had the largest proportion in domain 24 of both anterior and posterior slices. Moreover, domain 12 and domain 13 were enriched in L5 PT and L5 IT cells. Domains 7 and 8 were anterior‐specific clusters, which was the main olfactory bulb region according to annotation. These two domains were enriched by Meis2 and Sperpinf1. A previous study showed that Meis2 plays an important role in adult olfactory bulb (OB) neurogenesis.^[^ ^27^ ^]^ Moreover, we investigated each cell type for each method respectively (Figure 5d; Figure S14, Supporting Information). As illustrated in the spatial mapping of L2/3 IT, L4, L5 IT, and L6 CT of SMILE, IRIS, CARD, and Cell2location, we found that SMILE detected these cell types in a cleaner mode, while there were large amounts of false positive spots detected by the other three methods. This observation was further confirmed by examining the well‐known cell type specific marker genes such as Lamp5, Calb1, Rorb, and Cplx3, where these genes were exclusively overexpressed in the regions detected by SMILE (Figure 5d). Together, SMILE is robust, as demonstrated by either the expression of marker genes or the anatomy of the brain, and can establish an agreement between gene expression‐based clustering and anatomical annotation, providing a more thorough and comprehensive understanding of spatial heterogeneity than can be achieved through visual inspection.

### 2.6 SMILE Pinpoints the Key Altered Cellular Microenvironments in Human Psoriasis

**Figure 6:**
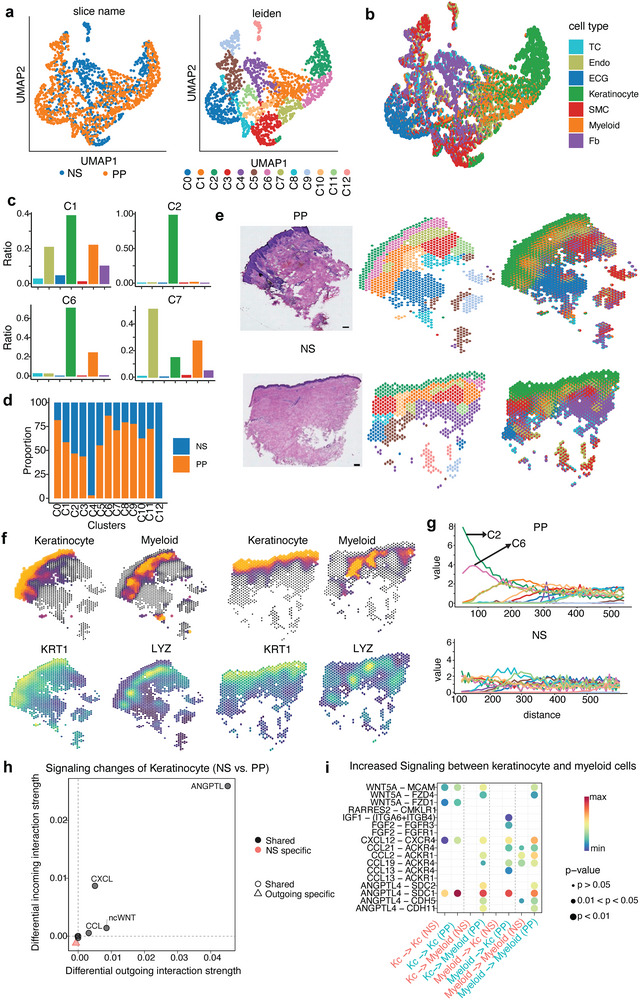
SMILE reveals differential cellular compositions and microenvironment signaling between healthy and psoriatic skin. a) UMAP visualization of the integrated embedding data from SMILE. Each point represents one spot. Spots are colored by biological conditions (left) and by identified spatial domains. b) UMAP visualization of the data and each point is represented by a pie chart showing the cell type compositions. c) Bar plot of the average ratios of each cell type in the domains C1, C2, C6, and C7. d) Bar plot showing the proportions of spots coming from NS or PP for each spatial domain. e) H&E staining of the NS and PP biopsies used for spatial sequencing (left), and spatial plot of spots colored by spatial domains (middle) and cell type compositions (right). f) Visualization of spatial distribution of the keratinocyte and myeloid cells detected by SMILE for NS and PP (top). The bottom shows the spatial plots showing the denoised expression levels of KRT1 and LYZ in NS and PP samples. g) Visualization of the co‐occurrence scores of domain 2 for PP (top) and NS (bottom) at increasing distance thresholds across the tissue. h) The differential outgoing and incoming interaction strength for keratinocytes. Signaling with positive values indicates increased interaction strength in PP compared to NS. i) Dot plot of probabilities mediated by L‐R pairs between keratinocytes and myeloid cells.

Next, we tested SMILE's ability to identify the altered spatial heterogeneity across different biological conditions by using single cell and spatial transcriptomics data of healthy normal skin (NS) and psoriatic skin (PP).^[^ ^28^ ^]^ SMILE identified 12 spatial domains and revealed the cell type compositions within each spot to understand the cellular heterogeneity of spatial domains (**Figure** **6**a,b). Keratinocytes were enriched in the domains C1, C2, C6, and C7 (Figure 6c). Myeloid cells were located in domains C1, C6, and C7. Spots in domains C1 and C2 were enriched in both normal and psoriatic skin, while the spots in domains C6 and C7 were predominantly enriched in psoriatic skin (Figure 6d), indicating the highly enriched immune cells in PP compared to NS, which is consistent with the fact that psoriatic disease is an immune‐mediated inflammatory condition. As expected, keratinocytes were located in the epidermis of both NS and PP. Myeloid cells, T cells (TC), and endothelial cells (Endo) were located in the superficial dermis in proximity to the epidermis, whereas fibroblasts (Fb), smooth muscle cells (SMC), and eccrine gland cells (ECG) were located deeper in the dermis (Figure 6e).

To further investigate the immunopathogenesis of psoriasis, we focused on the interaction between keratinocytes and myeloid cells deconvoluted by SMILE. Consistent with the spatial expression distributions of known epidermal marker genes like KRT1^[^ ^29^ ^]^ and myeloid marker genes like LYZ,^[^ ^30^ ^]^ keratinocytes and myeloid cells were located in the skin tissue in a clear manner (Figure 6f). The domain C2 was enriched with keratinocytes and domain C6 was enriched with both keratinocytes and myeloid cells. Co‐occurrence analysis further showed that domain C2 is co‐enriched at short distances with domain C6 in psoriatic skin compared to normal skin (Figure 6g), suggesting possible interactions between keratinocytes and myeloid cells. To study the signaling mechanisms in the pathogenesis of psoriasis, we utilized CellChat^[^ ^31^ ^]^ to systematically infer cell‐cell communication between keratinocytes and myeloid cells. We observed increased communication strengths of signaling pathways in PP compared to NS, including ANGPTL, CXCL, ncWNT and CCL (Figure 6h). CXCL and CCL are well‐known inflammatory signaling pathways that can promote the migration of immune cells into the keratinocytes. Non‐canonical Wnt (ncWNT) signaling like Wnt5a is found to stimulate keratinocyte proliferation and secretion of inflammatory cytokines.^[^ ^32^ ^]^ Recent studies reveal that ANGPTLs function in angiogenesis, lipid and energy metabolism and regulation of inflammation.^[^ ^33^, ^34^ ^]^ Further investigation of the ligand‐receptor (L‐R) pairs contributing to the cell‐cell communication between keratinocytes and myeloid cells revealed uniquely enriched signaling in PP compared to NS (Figure 6i). Specifically, WNT5A, CXCL12, CCL2, CCL19, and ANGPTL4 are enriched signaling in PP that send signals from keratinocytes to myeloid cells. In the other signaling direction from myeloid cells to keratinocytes, the signaling IGF1, FGF2, CXCL12, CCL21, CCL19, CCL13, and ANGPTL4 are enriched in PP. The appearance of CXCL12, CCL19, and ANGPTL4 in both directions indicates the positive feedback signaling between keratinocytes and myeloid cells, which likely amplifies the pathogenesis of the psoriasis. These results are consistent with previous observations that CXCL12 and ANGPTL4 regulate psoriasis via modulating hyperproliferation and inflammation of keratinocytes in psoriasis skin.^[^ ^35^, ^36^ ^]^ Taken together, through the multiscale analysis of spatial heterogeneity in skin tissue, SMILE is able to uncover the differential cellular compositions and microenvironments between healthy and psoriatic conditions.

### 2.7 SMILE Accurately Integrates Spatial Transcriptomic Data of Different Platforms in Both Spatial Domain and Cell Type Level

**Figure 7:**
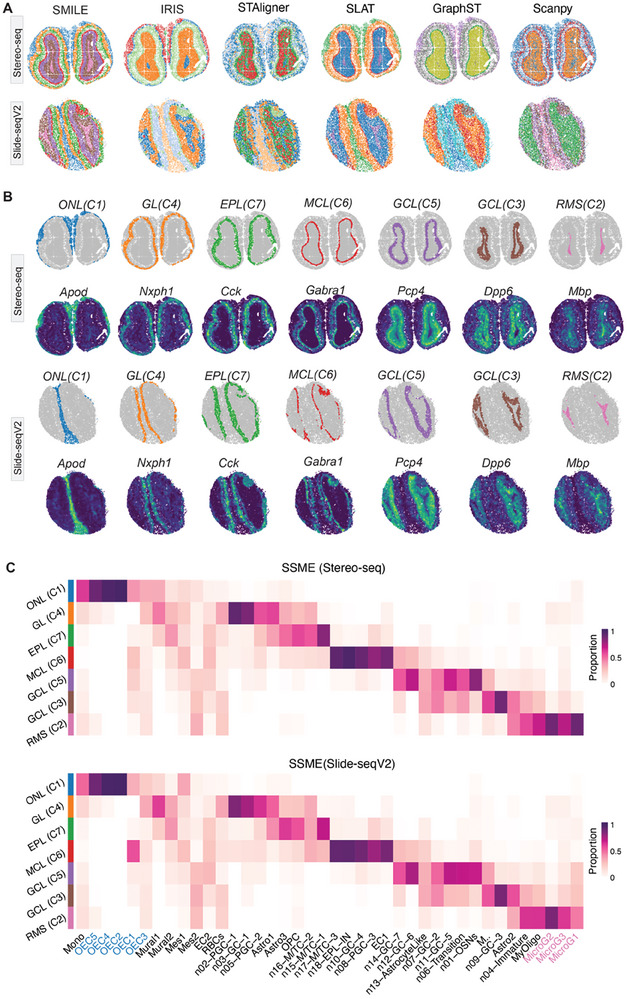
SMILE identifies spatial domains with cell type compositions on MOB data coming from Stereo‐seq and Slide‐seqV2 platforms. a) Spatial visualization of spatial domains identified in the integrated space of SMILE, IRIS, STAligner, SLAT, GraphST, and Scanpy, respectively. b) Spatial domains identified by SMILE and the corresponding marker genes in the slices of Stereo‐seq (top) and Slide‐seqV2 (bottom). c) Heatmap plot displaying the estimated mean cell type proportions for each cell type in each spatial domain detected by SMILE for Stereo‐seq (top) and Slide‐seqV2 (bottom), respectively.

Lastly, we applied SMILE to integrate the SRT of mouse olfactory bulb (MOB) coming from Stereo‐seq and Slide‐seqV2 platforms. SMILE outperformed other methods in delineating clear layer boundaries (**Figure** **7**a). IRIS, STAligner, GraphST, and Scanpy did not remove the batch effects completely (Figure 7a; Figure S18, Supporting Information). Then we investigated each cluster identified by mclust on the SMILE integrated space. The seven shared spatial domains in these two slices were spatially ordered from the outer to the inner layers of the MOB. Each cluster represented a structure annotated based on markers including the olfactory nerve layer (ONL), glomerular layer (GL), mitral cell layer (MCL), granule cell layer (GCL), and rostral migratory stream (RMS) (Figure 7b). SMILE accurately identified the elongated rostral migratory stream (RMS) within the mouse olfactory bulb slices, whereas STAligner, GraphST, and SLAT confused it with neighboring layers. Moreover, the denoised marker genes were accurately consistent with the distinct structures of the mouse olfactory bulb.

SMILE could reveal different spatial domains with differentially enriched cell types within the olfactory bulb at the same time (Figure 7c). Overall, layers of slices coming from Stereo‐seq and Slide‐seqV2 showed consistent cell type compositions. Specifically, Olfactory ensheathing cells (OECs) are the glial cells that reside primarily in the olfactory nerve layer, which is consistent with the previous study.^[^ ^37^ ^]^ Previous studies demonstrated that n15‐M/TC‐1, n16‐M/TC‐2, and n17‐M/TC‐3 strongly labeled cells in the mitral cell layer (MCL),^[^ ^38^, ^39^ ^]^ which could be revealed by SMILE. While IRIS, CARD, and Cell2location failed to detect such enrichment (Figures S19–S21, Supporting Information). n06‐Transition, n07‐GC‐2, n09‐GC‐3, n11‐GC‐5, n12‐GC‐6 and n14‐GC‐7 were located in the granule cell layer. n04‐Immature cells were residing in the rostral migratory stream. Moreover, microglial cells located in the RMS layer and were reported to maintain an environment permissive to neuroblast migration in the early postnatal RMS in the previous study.^[^ ^39^ ^]^

## 3 Discussion

A rapid increase in spatial genomics datasets collected from different slices and experiments presents a major challenge in aligning and comparing spatial datasets due to the low spatial resolution, particularly for the understanding of cellular heterogeneity and tissue organization. To understand how the cells are organized at multiscale levels from global spatial domains to local cell type heterogeneity, in this study, we presented a deep learning method, named SMILE, to integrate spatial transcriptomic data from multiple slices by taking scRNA‐seq data as a reference. SMILE can align different slices and reveal spatial heterogeneity at both spatial domain and cell type levels.

Unlike existing aligned methods like STALigner and SLAT, which were mainly designed for domain alignment and relied on marker genes to annotate the identified spatial domain. Many spatial datasets do not have single‐cell resolution, which prevents their application in revealing cellular compositions, especially when integrating slices from normal and disease samples. The recently proposed versatile method IRIS can integrate multiple spatial transcriptomics datasets and single cell reference data. However, it focuses on improving spatial domain identification with the help of single‐cell reference data. In agreement with the statement in the original study of IRIS,^[^ ^10^ ^]^ our results also showed that the deconvolution performance of IRIS was not good. GraphST can align spatial transcriptomics data of multiple slices and deconvolute spots of one slice by integrating with single‐cell reference data. However, these two tasks are separate. Moreover, it cannot deconvolute spots of multiple slices, which prevents the application in revealing disease‐specific cellular compositions and microenvironments. On the contrary, SMILE is able to facilitate the multi‐slice SRT analysis at multi‐scale levels ranging from molecular, cellular, to domain levels.

Using the simulated spatial dataset, SMILE has been shown to exhibit superior performance in slice alignment and spot cellular deconvolution. We demonstrated the capability of SMILE using three public datasets from different scenarios, including spatial transcriptomics of slices from the same donor, different donors and continuous regions as well as normal and disease conditions. Application of SMILE to the spatial transcriptomic data of human psoriasis skin uncovers disease‐specific cellular compositions and cell‐cell communications, which is important for understanding disease onset and progression.

While SMILE opens up new avenues for analyzing spatial transcriptomics at a multiscale level, its performance may be further improved. The current SMILE focuses on spatial coordinates and transcriptomics data without considering histological images. Future work is expected to incorporate such information to facilitate a more comprehensive understanding of cellular heterogeneity and tissue organization. Moreover, with the advance of spatial multi‐omics,^[^ ^40^, ^41^ ^]^ the framework of SMILE will likely be extended to other modalities of spatial genomics.

## 4 Experimental Section

### Data Preprocessing

SMILE takes gene expression profiles, spatial coordinates of multiple slices and single cell reference data as input. Each spatial gene expression dataset was stored in an *N × D* matrix of unique molecular identifier (UMI) counts with *N* spots and *D* genes, along with the (*x,y*) 2D spatial coordinates of each spot. The scanpy^[^ ^42^ ^]^ package was used to preprocess the data. Genes expressed in fewer than three spots were eliminated. The gene expression values in each spot were normalized such that the UMI count for each gene was divided by the total UMI count across all genes in each spot, multiplied by 10 000, and then transformed to a natural log scale with pseudocount equaling 1. Then two optimal feature selection methods were provided to select genes. On the one hand, high variable genes with top highest standardized variance across all spots were selected by sc.pp.highly_variable_genes with flavor = “seurat_v3.” On the other hand, spatial variable genes were selected by spatialDE^[^ ^43^ ^]^ with *q_value* being less than 0.01.

### Building Spatial Graph for Spatial Transcriptomic Data and Neighbor Graph for Single‐Cell Reference Data

SMILE uses the spatial coordinates of spots to build an undirected graph *A* using the squidpy package.^[^ ^44^ ^]^ The edges were built by an alpha‐complex‐based method. Specifically, for each spot *s*, its 1‐skeleton was computed using *V* (*s*) = {‖*x* − *s*‖ ≤ ‖*x* − *v*‖, ∀*v* ∈ *C*} , where *C* is the set of coordinates for all spots. Then a radius *r* was estimated by the mean distance of *k* nearest neighbors of the spot based on spatial coordinates. Spots in the following set *S* are connected, *S*  = {(*i*, *j*)|∩_*s* ∈ {*i*, *j*}_(*V*(*s*))∩*R*(*s*,  *r*)} . For single‐cell reference data, a shared nearest neighbor (SNN) graph was built using the scanpy package.^[^ ^20^ ^]^ This SNN graph was constructed by calculating the k‐nearest neighbors (20 by default) for cells based on the first 40 principal components after performing principal component analysis (PCA) on the feature matrix. The fraction of shared nearest neighbors between the spot and its neighbors was then used as weights of the SNN graph *A_sc_*.

### Extracting Low‐Dimensional Embeddings with Aggregating Features Over Neighboring Spots

To extract features that considered both spatial similarity and expression information, t a *k*‐layer Graph Convolutional Network (GCN) was adopted with the learned graphs *G_t_* (*X_t_*,*A_t_*) as input. Each graph convolutional layer aggregates features over neighboring nodes with a parametric rectified linear unit (RELU) as the activation function. The graph convolutional layer is $Z_{t}^{l}=GCN\;\left(Z_{t}^{l-1},A_{t}\right)={\hat{A}}_{t}\;Z_{t}^{l-1}\Theta$, where ${\hat{A}}_{t}={D_{t}}^{-1/2}\;\left(A_{t}+I\right){D_{t}}^{-1/2}.$ Inspired by a previous study,^[^ ^9^ ^]^ a contrastive learning strategy was used to capture both local information on the spatial neighbor graph *G_t_*. Specifically, a corruption graph ${\tilde{G}}_{t}=\left({\tilde{X}}_{t},{\tilde{A}}_{t}\right)\;,$ was generated which maintains a similar structure but randomly permute the gene expression. By the GCN method, an embedding *Z_t_* is generated on *G_t_*, another embedding $\tilde{Z_{t}}$ is generated on ${\tilde{G}}_{t}$. The default latent dimension size for low‐dimensional embeddings was set to *50*. To capture the global information, a summary vector *s* was computed by averaging *Z_t_*. To capture the local context of a spot *i*, a local vector *g_i_* was computed by a sigmoid of the mean of the representations of neighbors of spot *i*. The representation of spot *i* and local context vector *g_i_* form a positive pair, while the corresponding representation of spot *i* from the corrupted graph and the local context vector *g_i_* form a negative pair. The mutual information between $z_{i}^{t}$ and *g_i_* is larger than that of ${{\tilde{z}}_{i}}^{t}$ and *g_i_*. Mathematically, the corresponding learning process was achieved by maximizing the following two loss functions:

(1) $$\begin{array}{ccc} L_{local} & = & \sum_{t=1}^{T}\frac{1}{2N_{t}}\;\sum_{i=1}^{N_{t}}E_{\left(X_{t},A_{t}\right)}\left[logD\left(z_{i}^{t},g_{i}\right)\right]\\ & & +\;\sum_{t=1}^{T}\frac{1}{2N_{t}}\sum_{i=1}^{N_{t}}E_{\left(\tilde{X_{t}},\tilde{A_{t}}\right)}\left[log\left(1-D\left({{\tilde{z}}_{i}}^{t},g_{i}\right)\right)\right] \end{array}$$

where *D* is the discriminator and $D\;\left(z_{i}^{t},s\right)=Sigmoid\left({\left(z_{i}^{t}\right)}^{T}\Theta s\right),\;\Theta$ is the training weight. Moreover, assuming that the gene expression level of a spot was similar to its neighbor spots, a spatial coordinate constraint was added to enforce the spatial consistency in the embeddings. Mathematically, the constraint is formulated as follows:

(2) $$L_{R}=R\;\left(Z_{t}\right)=\sum_{i=1}^{N_{t}}\sum_{j=1}^{N_{t}}\frac{d_{i,j}^{c}*\left(1-d_{i,j}^{Z_{t}}\right)}{N_{t}*N_{t}}\;$$

where $d_{i,j}^{c}$ is the Euclidean distance between spot *i* to *j* on spatial coordinates, and $d_{i,j}^{Z_{t}}$ is the distance between spot *i* to *j* in embedding space. Finally, *Z_t_* is fed into a decoder to reverse them back into the gene expression space. Specifically, the decoder is defined as $H_{t}^{l}=GCN\;\left(H_{t}^{l-1},{\hat{A}}_{t}\right)={\hat{A}}_{t}\;H_{t}^{l-1}\Theta .$ The loss of the gene expression self‐reconstructed part is: $L_{recon}=\sum_{i=1}^{N}{∥x_{i}-h_{i}∥}_{F}^{2}$, where *x_i_* and *h_i_* are the normalized gene expression and reconstructed gene expression for spot *i*.

### Dissecting Spots with Low‐Dimensional Embeddings of Spatial Transcriptomic and Single‐Cell Reference Data

The contrastive graph learning mentioned above was treated as a feature extractor. The feature extractor module was first trained with spatial transcriptomics data, and then it was applied to the single‐cell reference data with the trained parameters to reduce the dimensions of the single‐cell reference data. Next, a classifier was built for the low‐dimensional embeddings of single cell and SRT data with the following loss:

(3) $$\begin{array}{ccc} L_{classifier}L_{sc} & & +\;\lambda \sum_{t=1}^{T}{∥X_{t}-P_{t}B^{T}∥}_{F}^{2}\\ & & +\;\gamma \left(\sum_{t=1}^{T}\sum_{i=1}^{N_{t}}\sum_{j=1}^{N_{t}}\frac{d_{i,j}^{P_{t}}*\left(1-d_{i,j}^{Z_{t}}\right)}{N_{t}*N_{t}}\sum_{t=1}^{T}\frac{d_{i,j}^{P^{a}}*\left(1-d_{i,j}^{Z_{a}}\right)}{N_{t}*N_{t}}\right) \end{array}$$

where *L_sc_* is the cross‐entropy loss on single‐cell referenced data. λ and γ are hyperparameters with default values equaling 1, which can be selected by splitting the spatial transcriptomics into training, validation and test data sets based on the *x*‐axis of spatial coordinates (Figure S23, Supporting Information). Each row of *P_t_* represents the probabilities of the *K* cell type assignments to each spatial spot of slice *t*. *B* is the mean expression of each cell type with *D* genes across *K* cell types. *P^a^* is the probability of anchor pairs, which were identified by MNN on embeddings.

### Aligning Multiple Spatial Transcriptomic Datasets Based on Anchors

After learning the cell type assignments of the spatial profiles, the anchors across multiple slices were detected using the MNN (mutual nearest neighbor) method on the assignments, which indicated anchors should have similar cellular compositions. Then spatial transcriptomic datasets coming from any pair of conditions (e.g., slices, platforms, samples et al.) could be aligned together based on anchors to remove batch effects across different datasets, which might mask the actual biological signals. To achieve this goal, Maximum Mean Discrepancy (MMD) loss was adopted on anchors as follows:

(4)

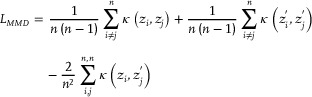

where *n* is the number of anchors and κ(·) is the Gaussian kernel. Finally, the cell type assignments of each slice were updated with the aligned embeddings.

### Semi‐Supervised Learning on Spatial Transcriptomic Data with Domain Annotation

If one slice has spatial domain information, SMILE could leverage such annotation information to achieve optimal performance. In SMILE, a classifier was adopted built using a three‐layer perceptron neural network with hidden dimensions of 512, 512, and a rectified linear unit (*ReLU*) as an activation function. The loss function is: $L_{semi}=-\frac{1}{m}\sum_{k=1}^{m}y_{k}\cdot log\left({\overset{⌣}{y}}_{k}\right)$where *m* is the number of spots that have labels. Finally, the trained classifier can be used to predict functional domains for other slices.

### Cell‐Cell Communication Analysis Using CellChat

Based on the deconvoluted cell types in normal and psoriasis skin, cell‐cell communication analysis was performed and the plots were generated by following the tutorial of CellChat v2^[^ ^45^ ^]^ on the analysis of spatially resolved transcriptomics data (https://github.com/jinworks/CellChat). The truncatedMean method with a trim value of 0.1 was used to calculate the average gene expression per cell group.

### Evaluation Metrics

To evaluate the performance of different methods in identifying spatial domains, ARI and NMI metrics were used.

### Adjusted Rand Index

Adjusted Rand Index (ARI) was introduced to determine whether real and predicted cell‐type clusters were similar to each other. The Rand Index (RI) computes similarity by considering all points identified within the same cell‐type cluster. The Adjusted RI (ARI) is the chance‐corrected version of the Rand index and is calculated using the RI as follows:

(5) $$ARI=\frac{RI-expected\;RI}{\max\left(RI\right)-expected\;RI}$$

The ARI value ranged from 0 to 1, with 0 indicating random labeling and 1 indicating perfect matching.

### Normalized Mutual Information

Normalized Mutual Information (NMI) is a variant of a common measure in information theory called Mutual Information. It is calculated as follows:

(6) $$NMI\left(U,V\right)=\frac{2\times I\left(U;V\right)}{H\left(U\right)-H\left(V\right)}$$

where *U* and *V* are categorical distributions for the real and predicted cluster annotations, *I* is the mutual information function and *H* is the Shannon entropy function.

### Data Availability

The datasets analyzed in this study were all from publicly available datasets. Specifically, 1) the spatial data of the human dorsolateral prefrontal cortex (DLPFC), which was generated from the 10x Genomics Visium platform can be accessed in the spatialLIBD package (http://spatial.libd.org/spatialLIBD). The reference scRNA‐seq data of DLPFC, which has 78886 nuclei from 34 brain samples was generated in a previous study^[^ ^19^ ^]^ and it was downloaded from GEO using the accession number: GSE144136. 2) Two spatial data of mouse posterior brain and anterior brain generated by 10x Genomics were downloaded from https://www.10xgenomics.com/datasets/mouse‐brain‐serial‐section‐1‐sagittal‐anterior‐1‐standard‐1‐0‐0 and https://support.10xgenomics.com ^/^ spatial‐gene‐expression/datasets/1.0.0/V1_Mouse_Brain_Sagittal_Posterior. The reference scRNA‐seq data with 28706 cells in 29 classes was downloaded with the accession number: GSE115746. After removing obscure classes such as “'Batch Grouping,” there were 23 classes left. 3) Human spatial transcriptomic data of psoriasis skin was available at GEO accession GSE144240. The corresponding human scRNA‐seq reference data was available at GEO accession GSE144236. 4) The mouse olfactory bulb tissue (MOB) data generated by Stereo‐seq and Slide‐seqV2 platforms were downloaded from https://github.com/JinmiaoChenLab/SEDR_analyses and https://singlecell.broadinstitute.org/single_cell/study/SCP815, respectively. The scRNA‐seq reference data of the mouse olfactory bulb was downloaded from GEO using the accession number: GSE121891.

## Conflict of Interest

The authors declare no conflict of interest.

## Author Contributions

Y.C. and C.Z. contributed equally to this work. L.Z. conceived the project. L.Z. and J.L. supervised the research. L.Z. developed and implemented the algorithm. L.Z., Y.C., C.Z. and Y.M. validated the methods. L.Z., J.L., Y.C. and C.Z. wrote the manuscript. All authors read and approved the final paper.

## Supporting information



Supporting Information

## Data Availability

The data that support the findings of this study are available from the corresponding author upon reasonable request.
