# Supplementary material for: Multiscale Dissection of Spatial Heterogeneity by Integrating Multi‐Slice Spatial and Single‐Cell Transcriptomics
Source: Adv Sci (Weinh). 2025 Feb 25;12(15):2413124. doi: 10.1002/advs.202413124 (PMC12005799; doi:10.1002/advs.202413124)
Supplement: Supplementary file 1 — Supporting Information [file ADVS-12-2413124-s001.pdf]

ADVANCED SCIENCE
Open Access

Supporting Information

for *Adv. Sci.*, DOI 10.1002/advs.202413124

Multiscale Dissection of Spatial Heterogeneity by Integrating Multi-Slice Spatial and Single-Cell Transcriptomics

Yuqi Chen, Caiwei Zhen, Yuanyuan Mo, Juan Liu and Lihua Zhang**

Supplementary for

Multiscale dissection of spatial heterogeneity by integrating multi-slice spatial and single-cell transcriptomics

Yuqi Chen[1#], Caiwei Zhen[1#], Yuanyuan Mo[1], Juan Liu[1*] and Lihua Zhang[1*]

[1] School of Computer Science, Wuhan University
[#]Authors contributed equally to this work
*Co-corresponding authors: liujuan@whu.edu; zhanglh@whu.edu.cn

Supplementary Figures

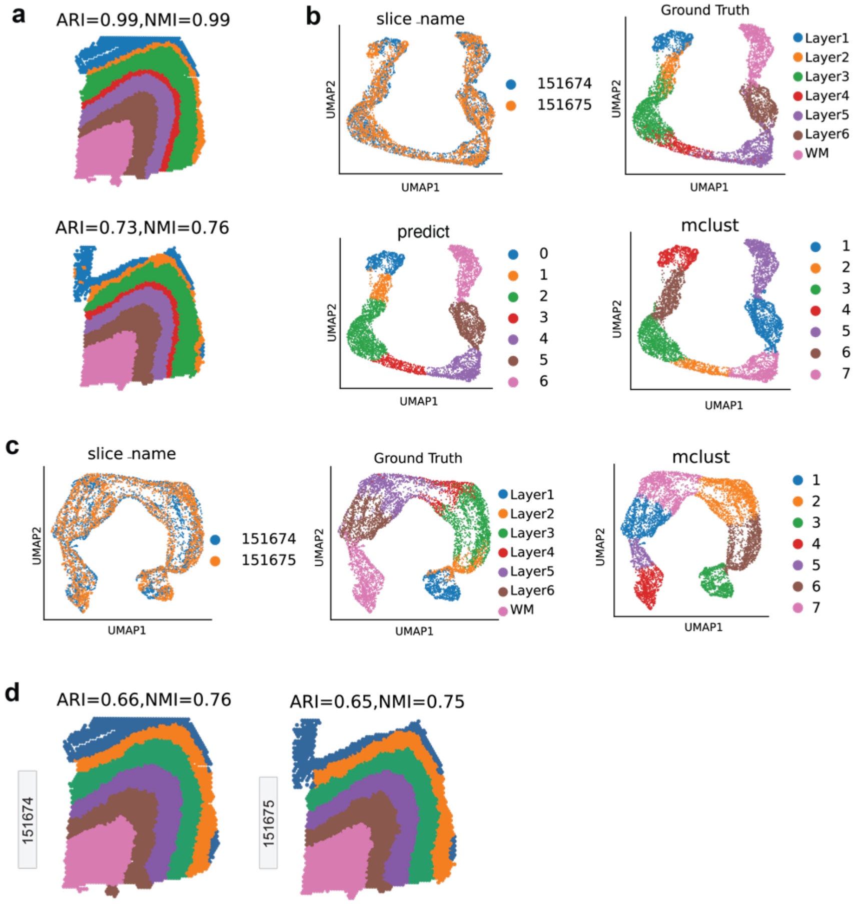

Figure S1. Evaluating alignment and domain prediction performance of SMILE_semi and SMILE on 151674 and 151675 slices of DLPFC datasets. a) Spatial visualization of predicted spatial domains by SMILE_semi on the slices of two DLPFC samples (151674 and 151675). b) UMAP visualization of the low-dimensional embeddings obtained by SMILE_semi. Spots are colored by slice_name, Ground Truth, prediction and mclust, respectively. c) UMAP visualization

of the low-dimensional embeddings obtained by SMILE. Spots are colored by slice_name, Ground Truth, and mclust, respectively. d) Spatial visualization of the spatial domains identified by mclust on the embeddings of SMILE with cluster number equaling 6.

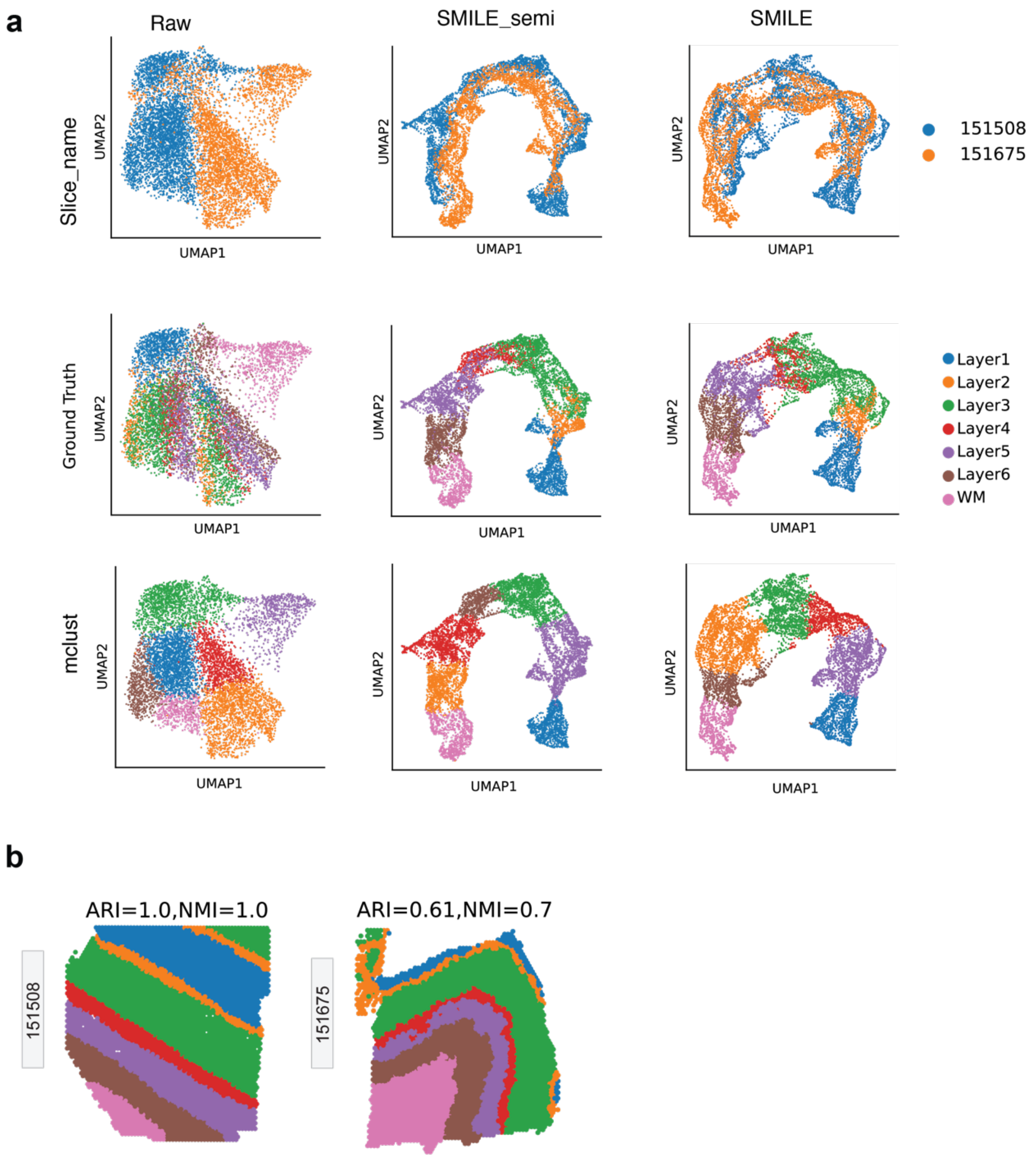

Figure S2. Evaluating alignment and domain prediction performance of SMILE_semi and SMILE on 151508 and 151675 slices of DLPFC datasets. a) UMAP visualization of the low-dimensional embeddings obtained by Raw data (left), SMILE_smi (middle) and SMILE (right). Spots are colored by slice_name, Ground Truth, and mclust, respectively. b) Spatial visualization

of predicted spatial domains by SMILE_semi on the slices of two DLPFC samples (151508 and 151675).

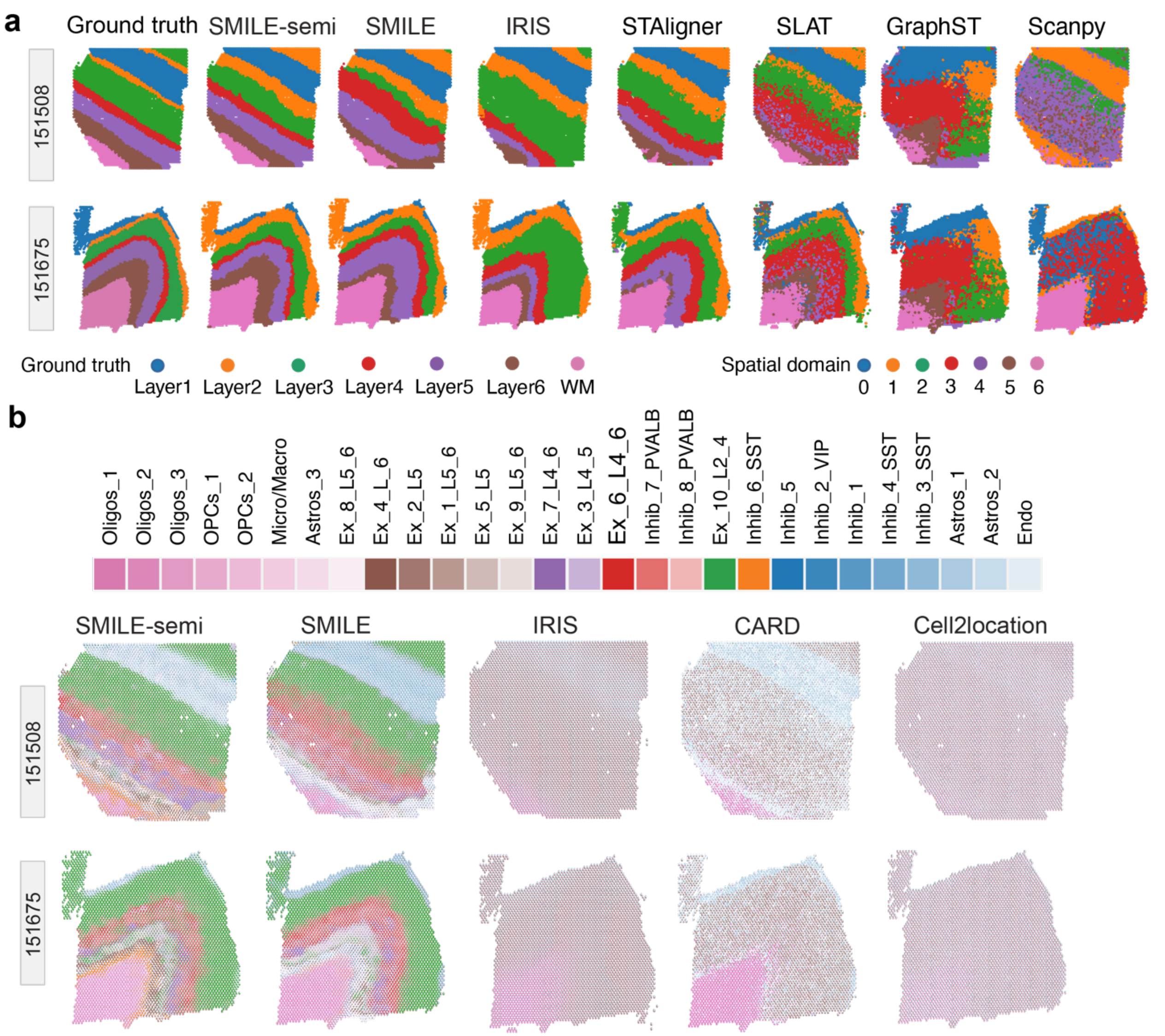

Figure S3. Evaluating domain identification and cell type deconvolution performance of different methods on 151508 and 151675 slices of DLPFC datasets. a) Spatial visualization of the domains of ground truth and those identified by SMILE_semi, SMILE, IRIS, STAligner, SLAT, GraphST and Scanpy. b) Mapping the deconvoluted cell type proportions by five methods onto tissues on the slices 151508 and 151675. Spatial scatter pie plot displays cell type proportions within spots.

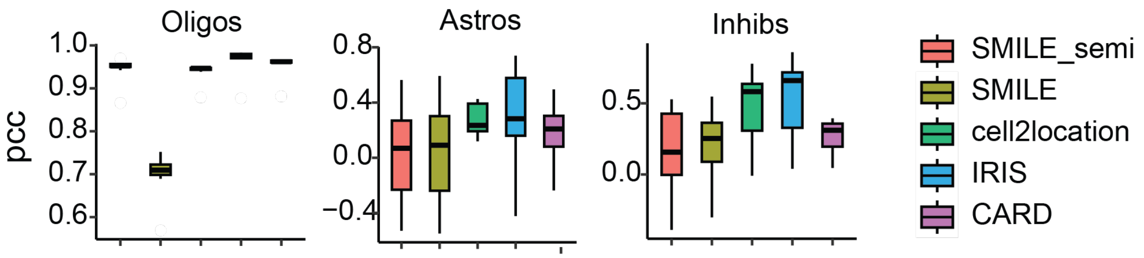

Figure S4. Boxplots showing Pearson correlation coefficients between the cell type proportions and the expression of marker genes of each major cell type.

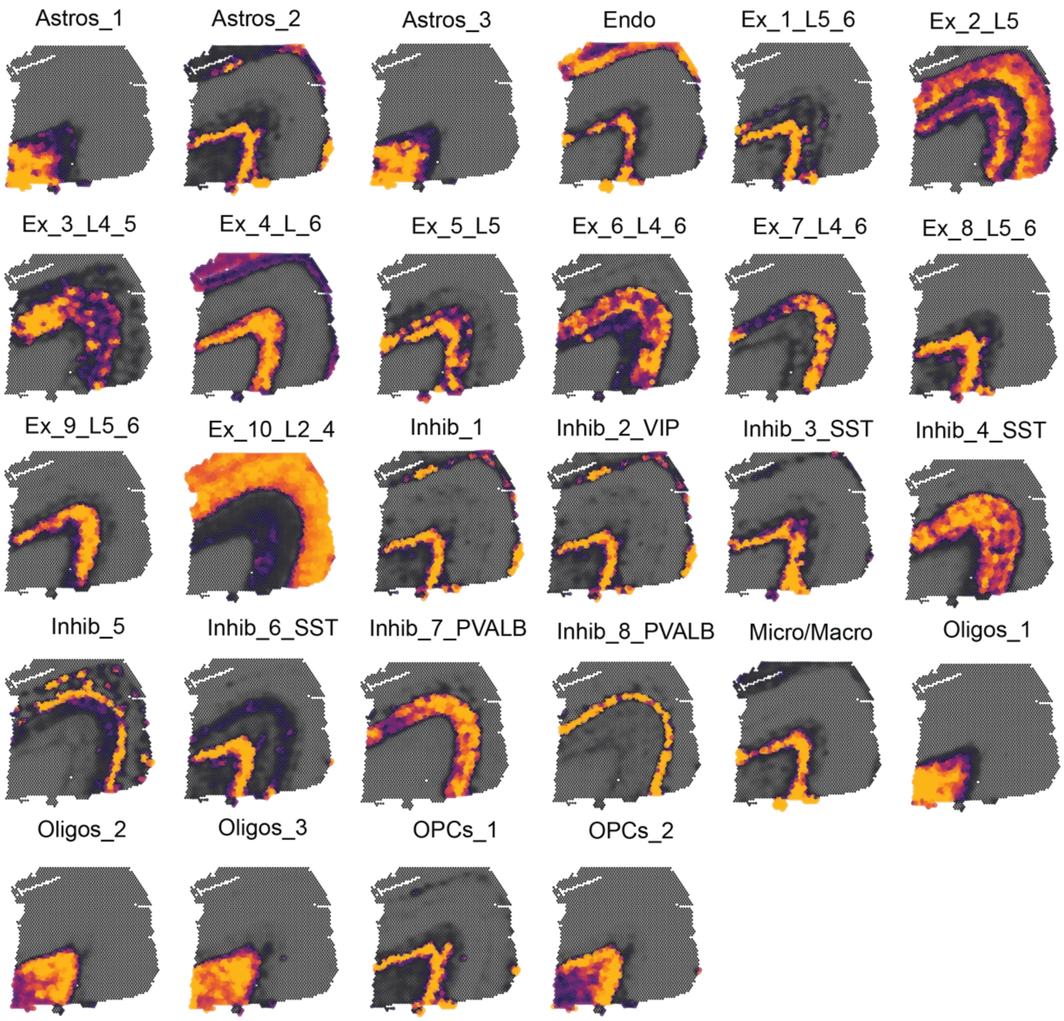

Figure S5. A spatial scatter plot displaying the spatial distribution of the cell type proportions of indicated cell types across spatial locations, which are inferred by SMILE_semi on slice 151674.

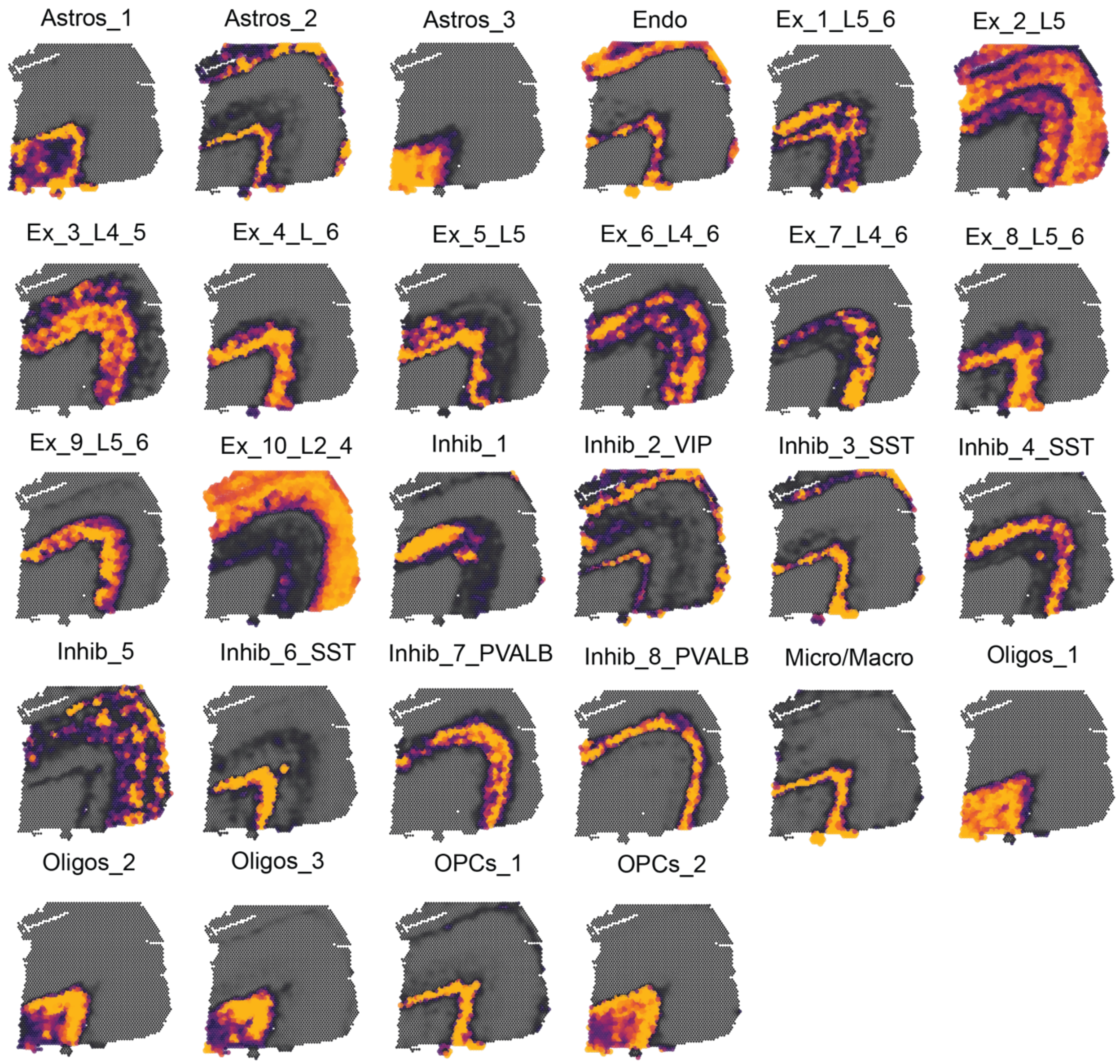

Figure S6. A spatial scatter plot displaying the spatial distribution of the cell type proportions of indicated cell types across spatial locations, which are inferred by SMILE on slice 151674.

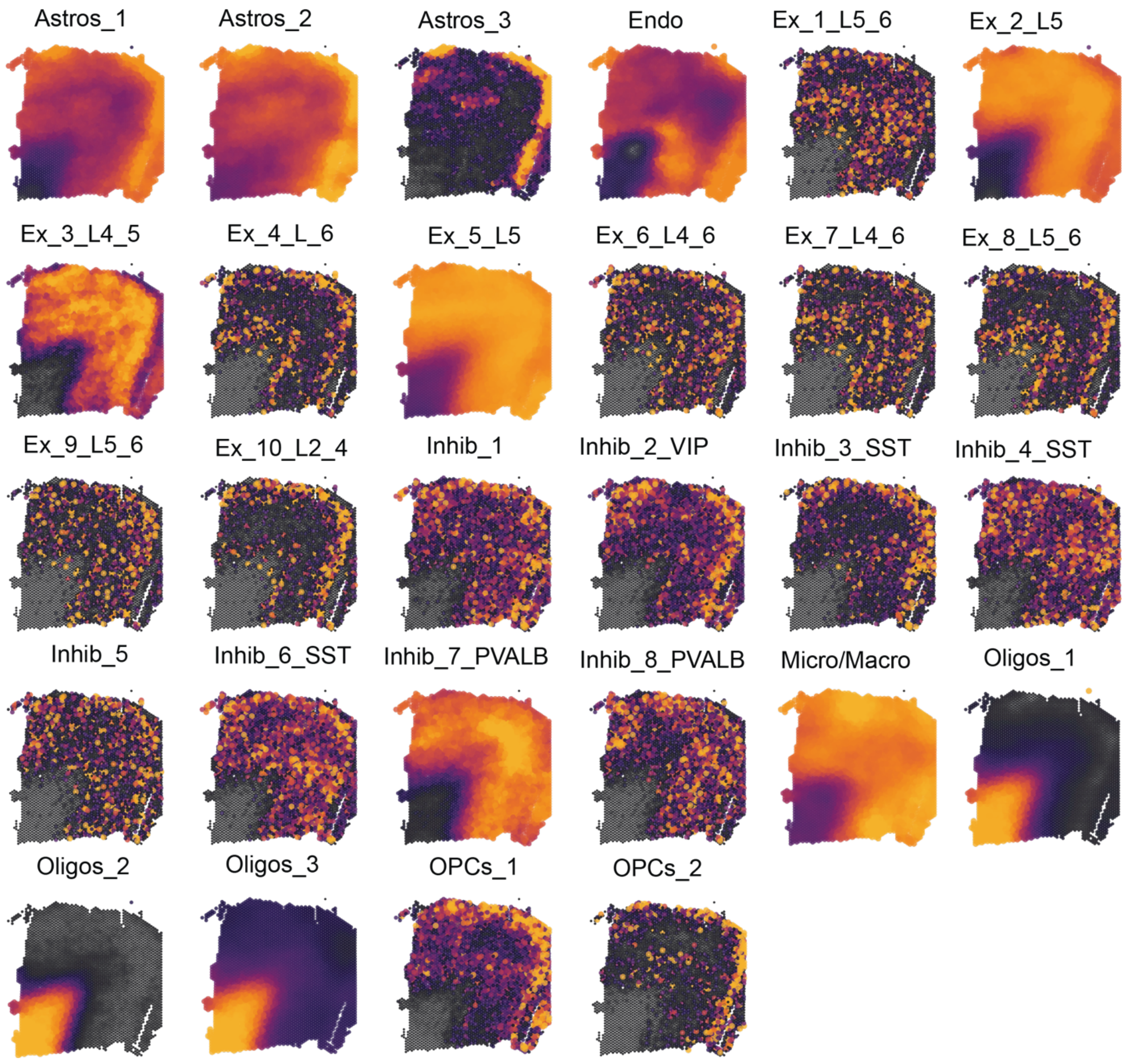

Figure S7. A spatial scatter plot displaying the spatial distribution of the cell type proportions of indicated cell types across spatial locations, which are inferred by IRIS on slice 151674.

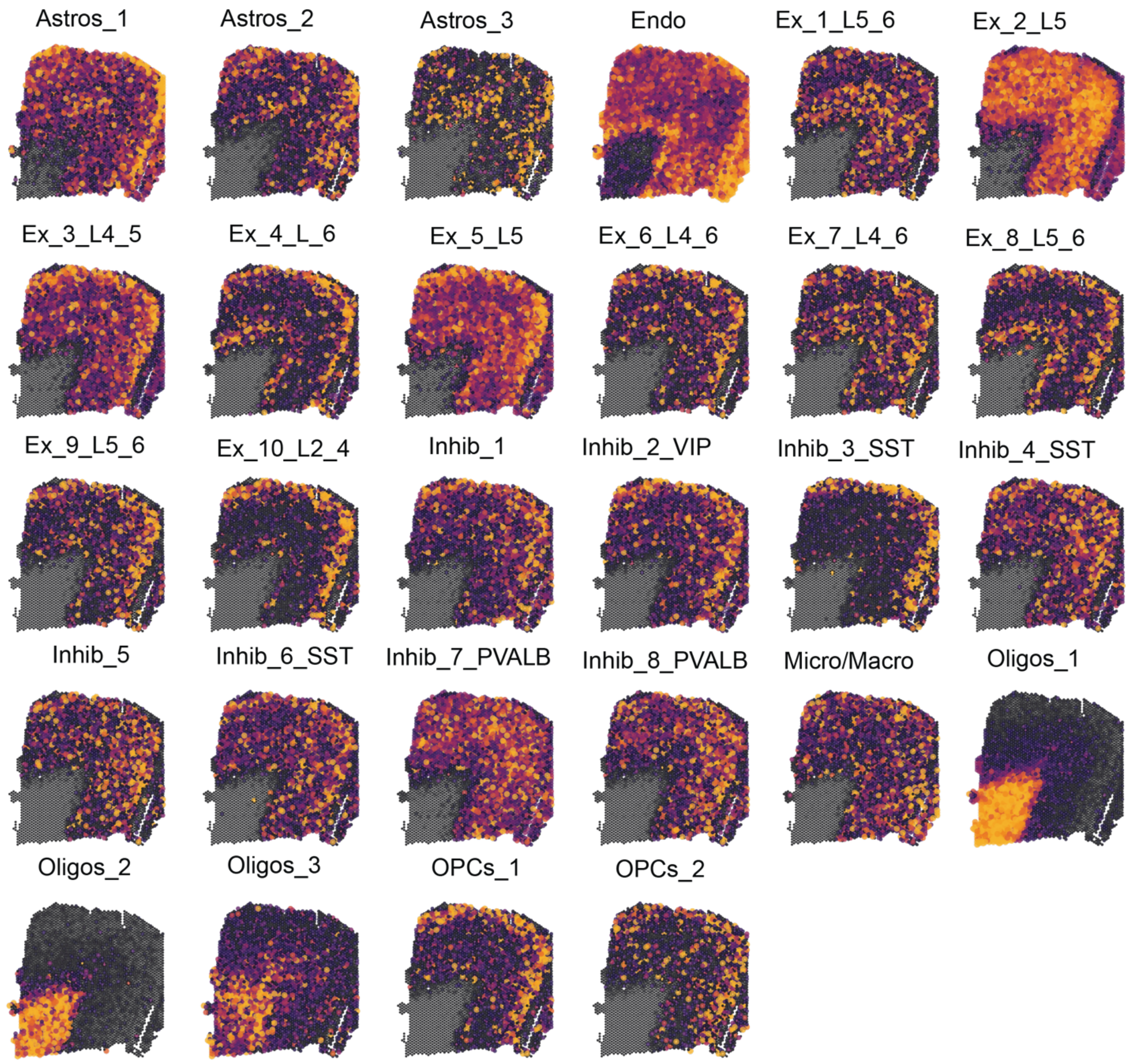

Figure S8. A spatial scatter plot displaying the spatial distribution of the cell type proportions of indicated cell types across spatial locations, which are inferred by CARD on slice 151674.

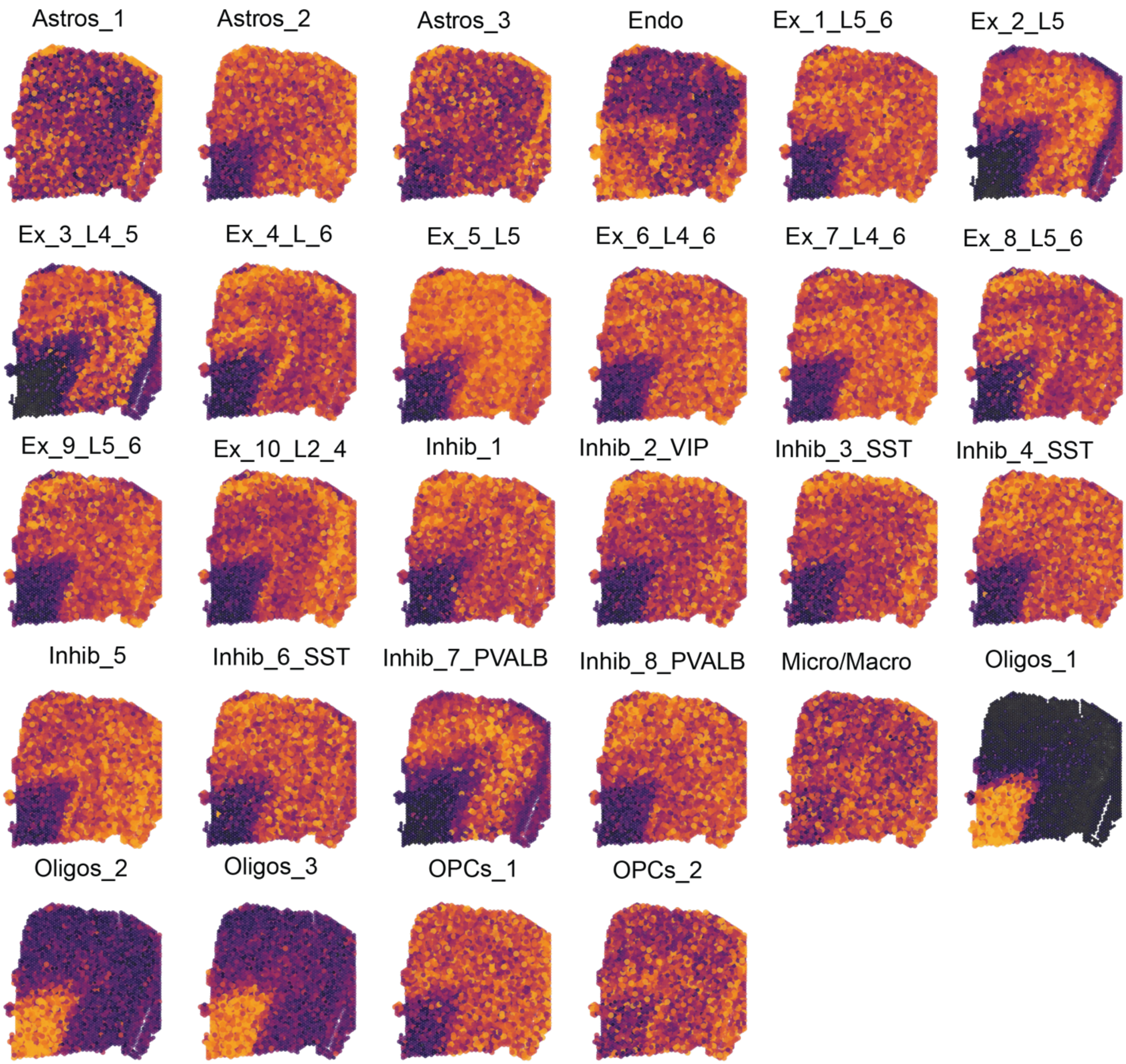

Figure S9. A spatial scatter plot displaying the spatial distribution of the cell type proportions of indicated cell types across spatial locations, which are inferred by Cell2location on slice 151674.

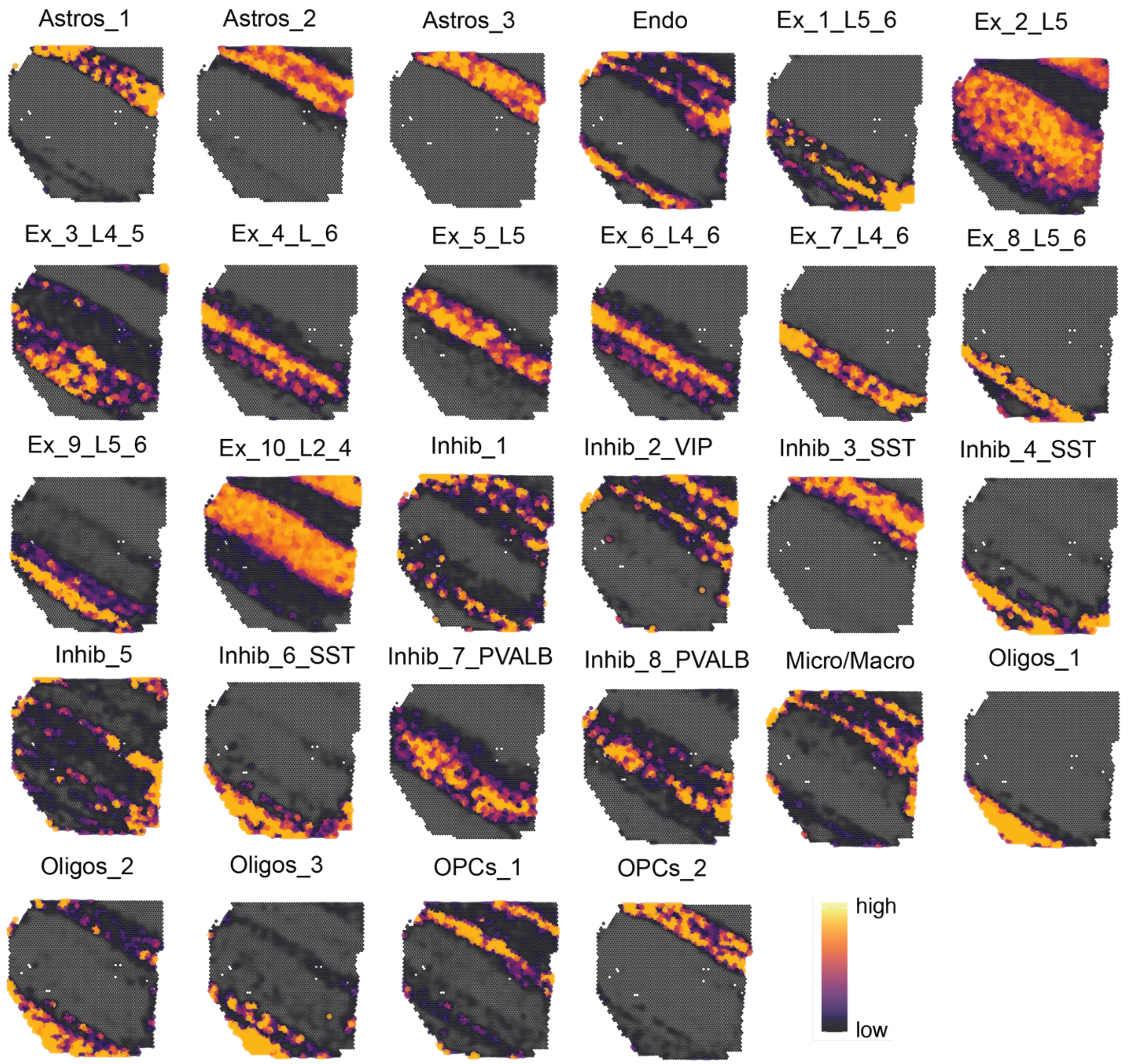

Figure S10. A spatial scatter plot displaying the spatial distribution of the cell type proportions of indicated cell types across spatial locations, which are inferred by SMILE on slice 151508.

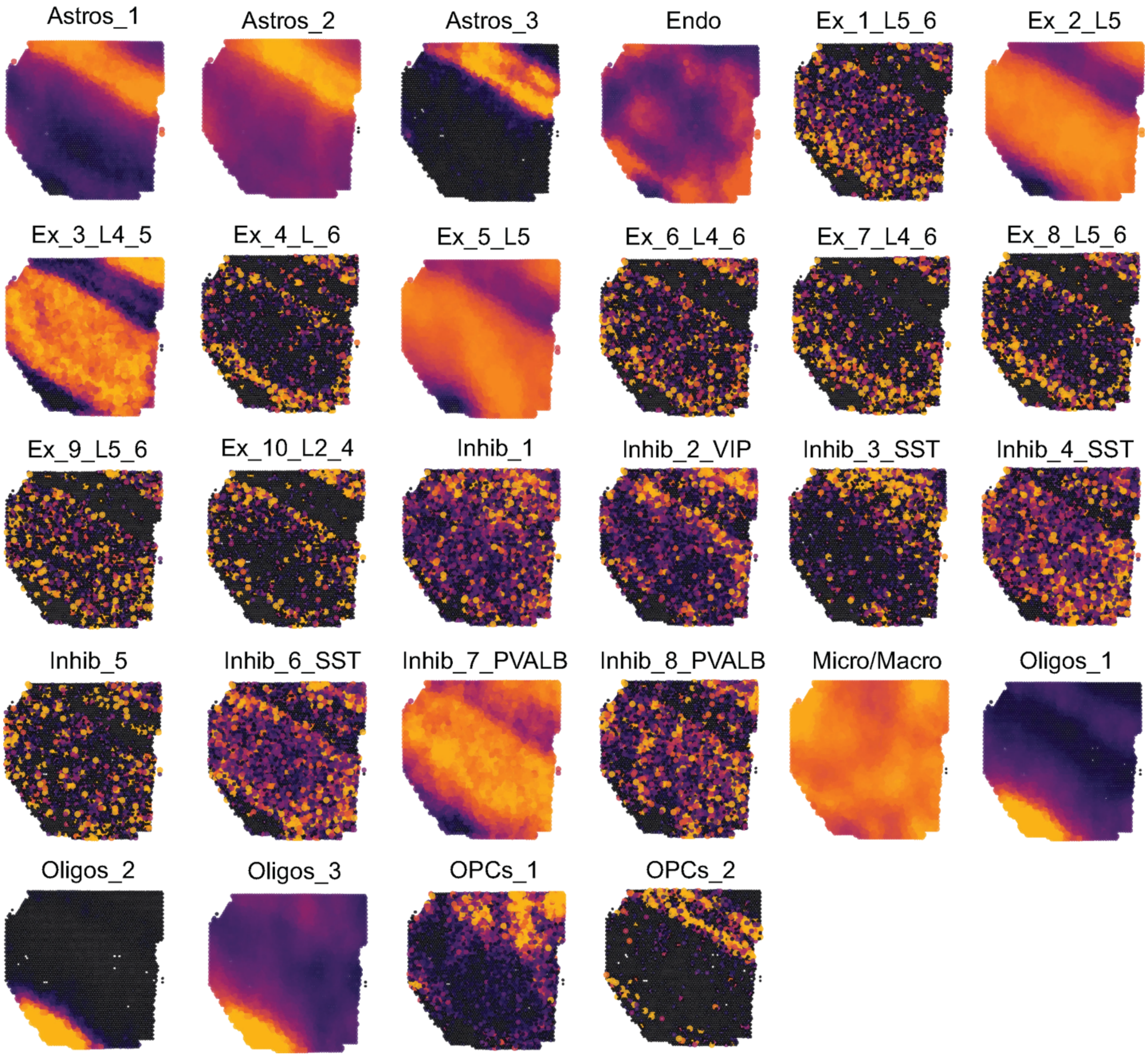

Figure S11. A spatial scatter plot displaying the spatial distribution of the cell type proportions of indicated cell types across spatial locations, which are inferred by IRIS on slice 151508.

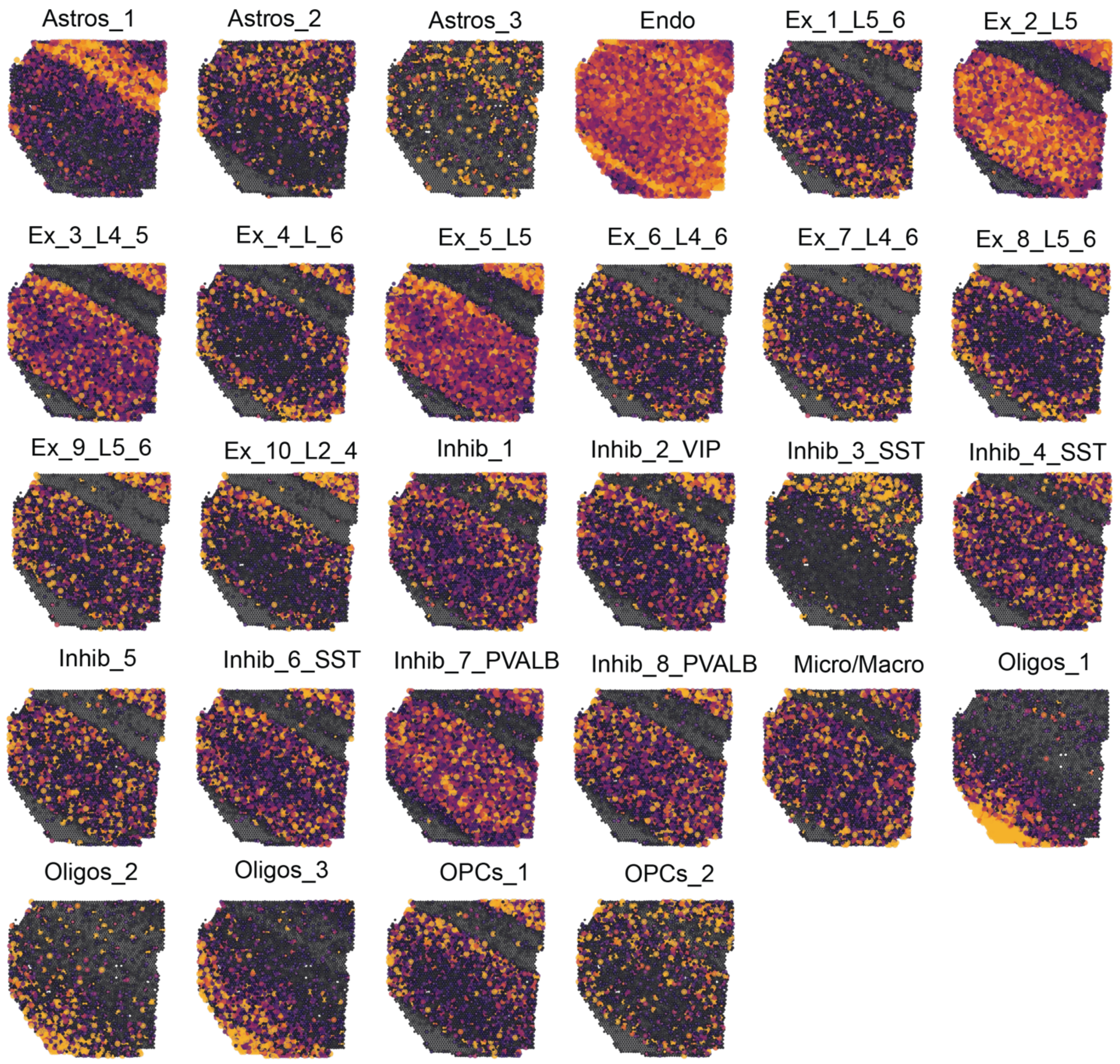

FigureS12. A spatial scatter plot displaying the spatial distribution of the cell type proportions of indicated cell types across spatial locations, which are inferred by CARD on slice 151508.

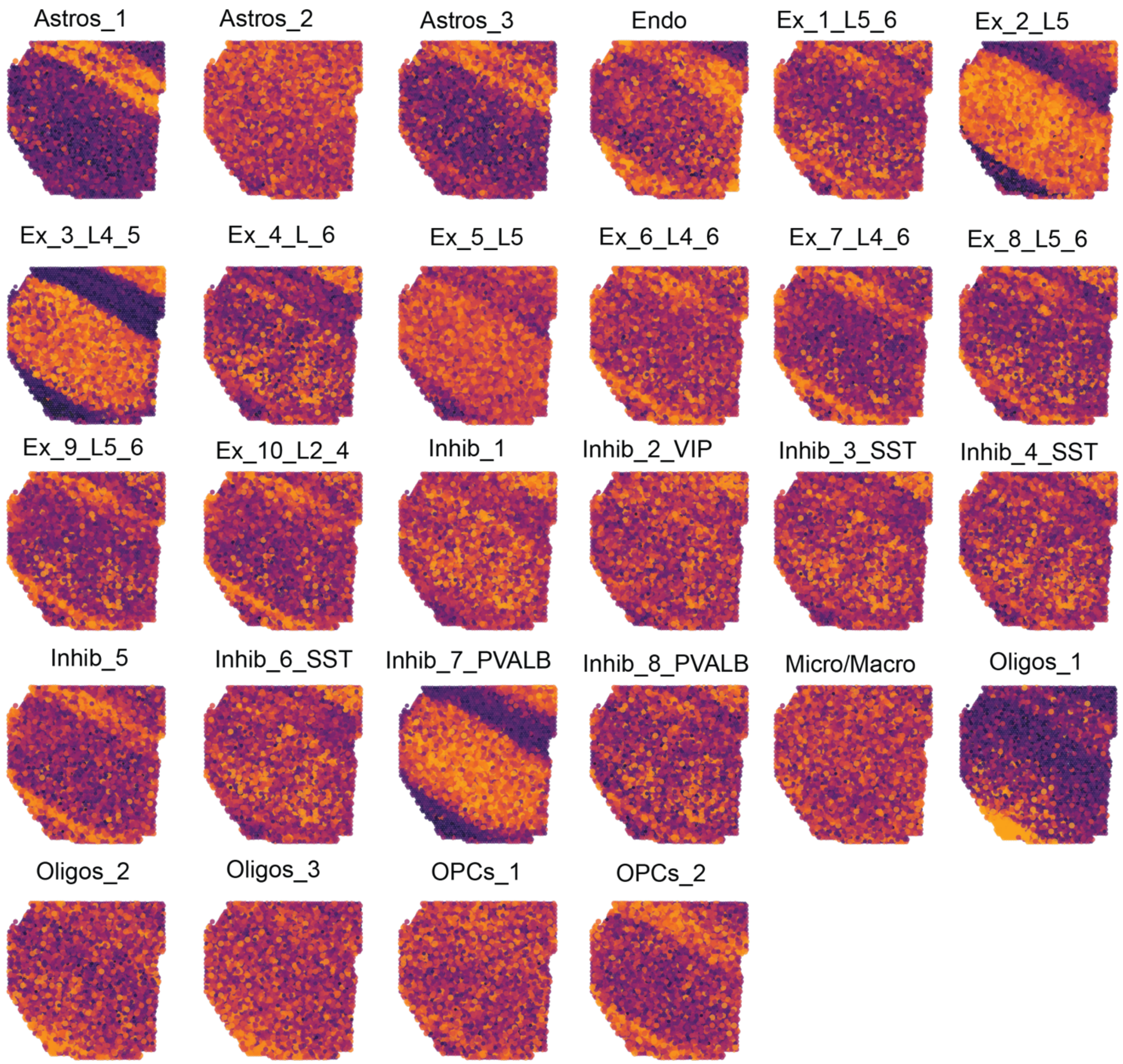

Figure S13. A spatial scatter plot displaying the spatial distribution of the cell type proportions of indicated cell types across spatial locations, which are inferred by Cell2location on slice 151508.

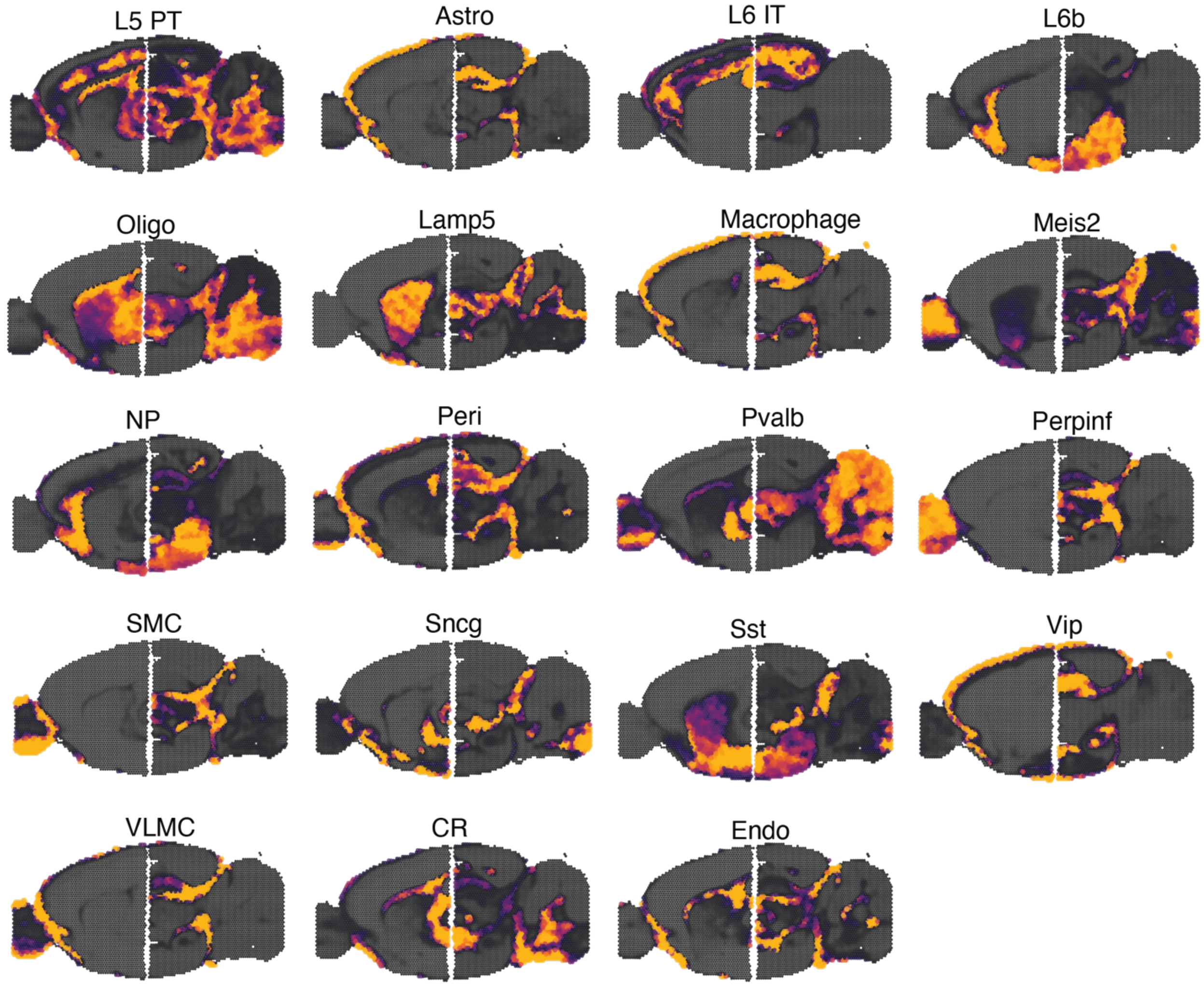

Figure S14. A spatial scatter plot displaying the spatial distribution of the cell type proportions of indicated cell types across spatial locations, which are inferred by SMILE on Mouse Brain data.

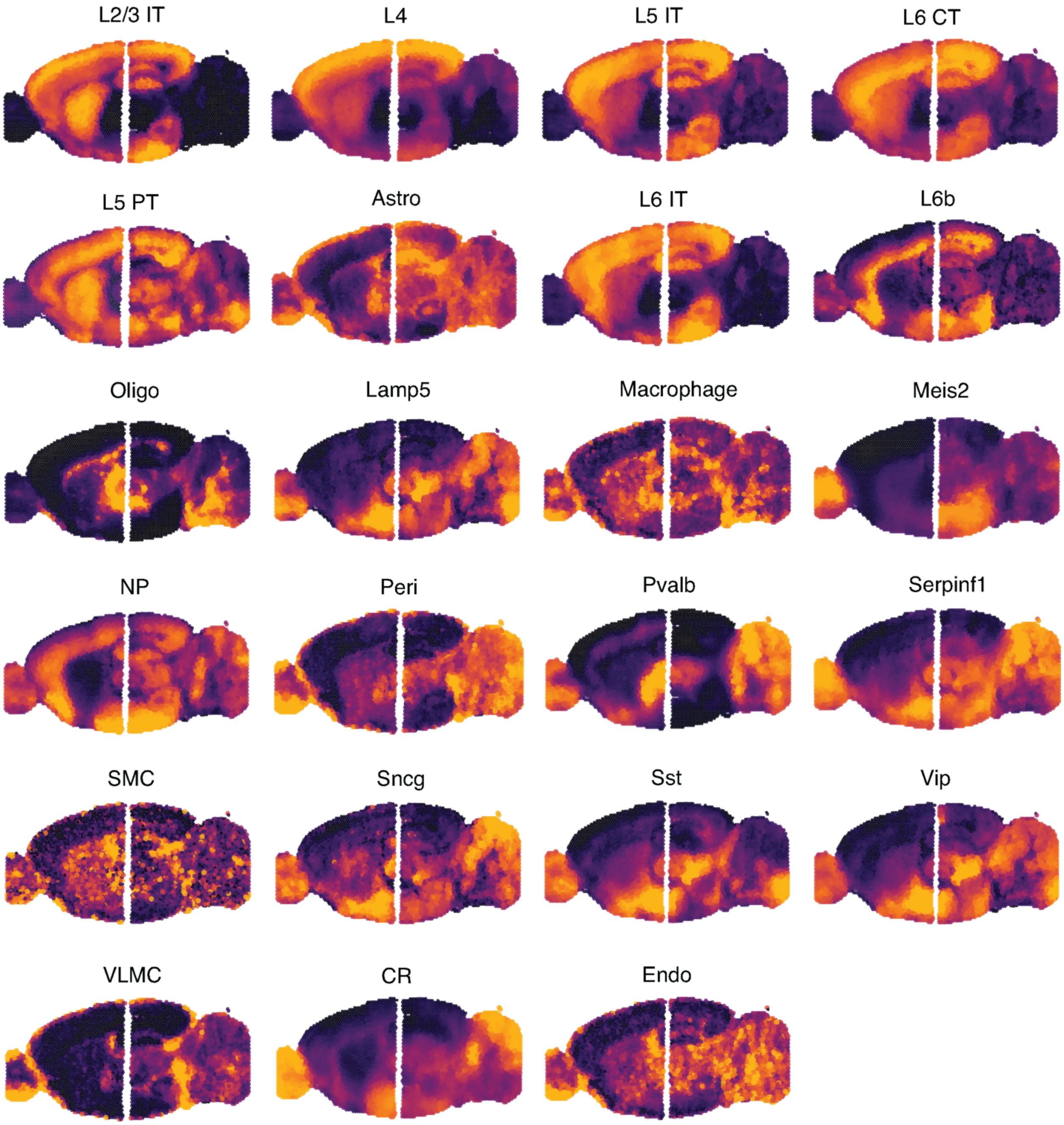

Figure S15. A spatial scatter plot displaying the spatial distribution of the cell type proportions of indicated cell types across spatial locations, which are inferred by IRIS on Mouse Brain data.

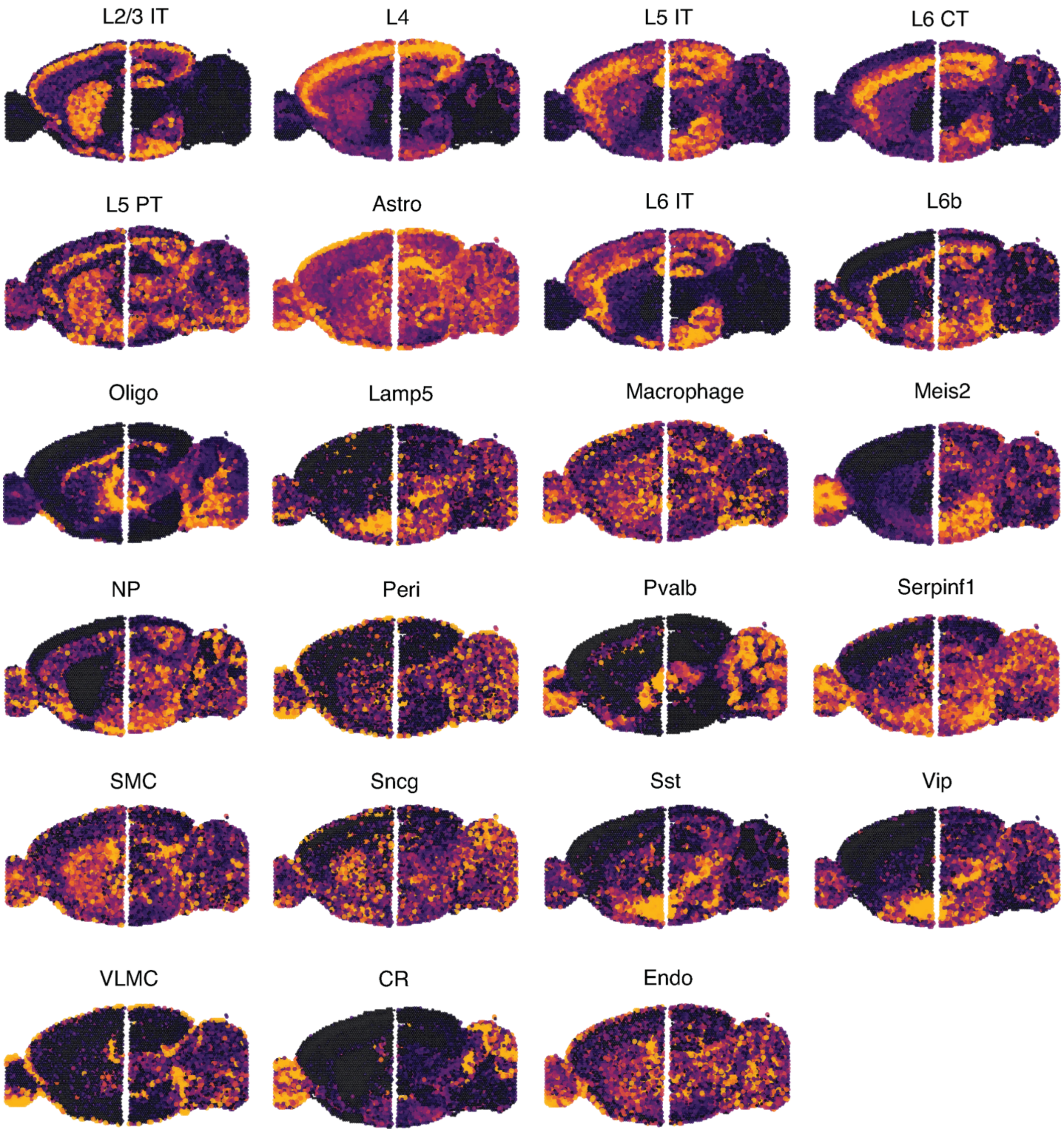

Figure S16. A spatial scatter plot displaying the spatial distribution of the cell type proportions of indicated cell types across spatial locations, which are inferred by CARD on Mouse Brain data.

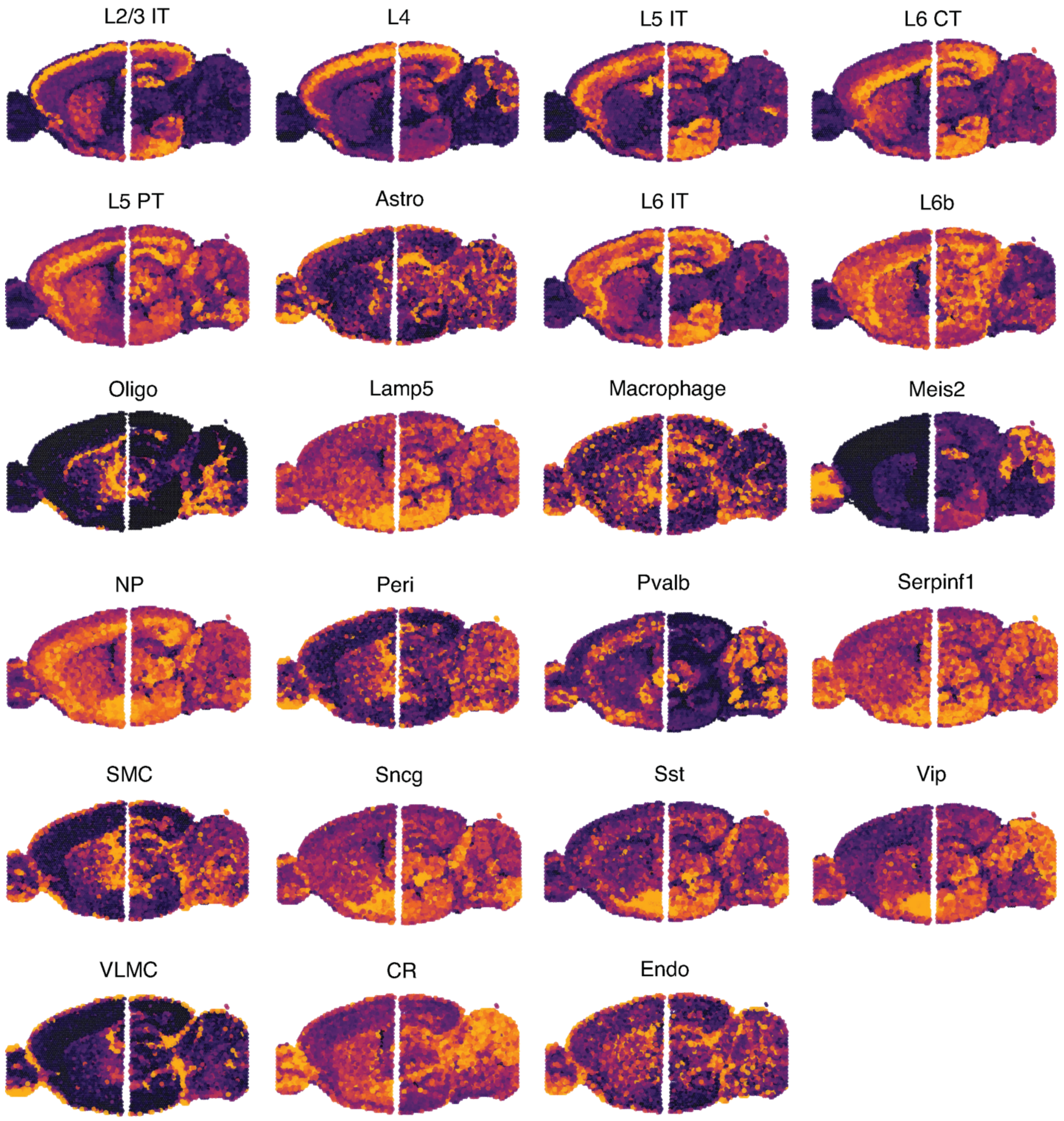

Figure S17. A spatial scatter plot displaying the spatial distribution of the cell type proportions of indicated cell types across spatial locations, which are inferred by Cell2location on Mouse Brain data.

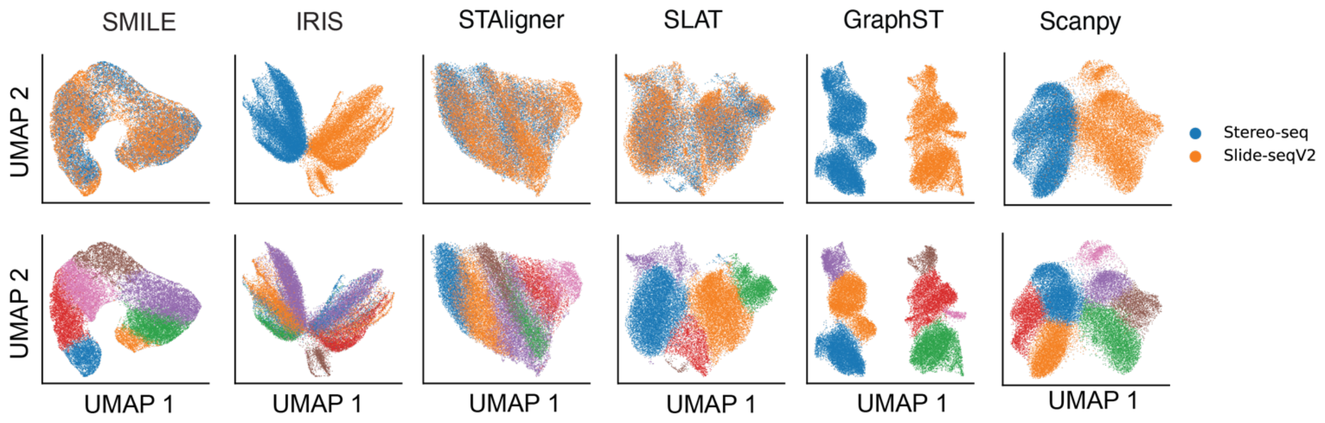

Figure S18. UMAP visualization of SMILE, IRIS, STAligner, SLAT, GraphST and Scanpy of MOB data generated from Stereo-seq and Slide-seqV2, colored by sequencing platforms (top) and clusters (bottom).

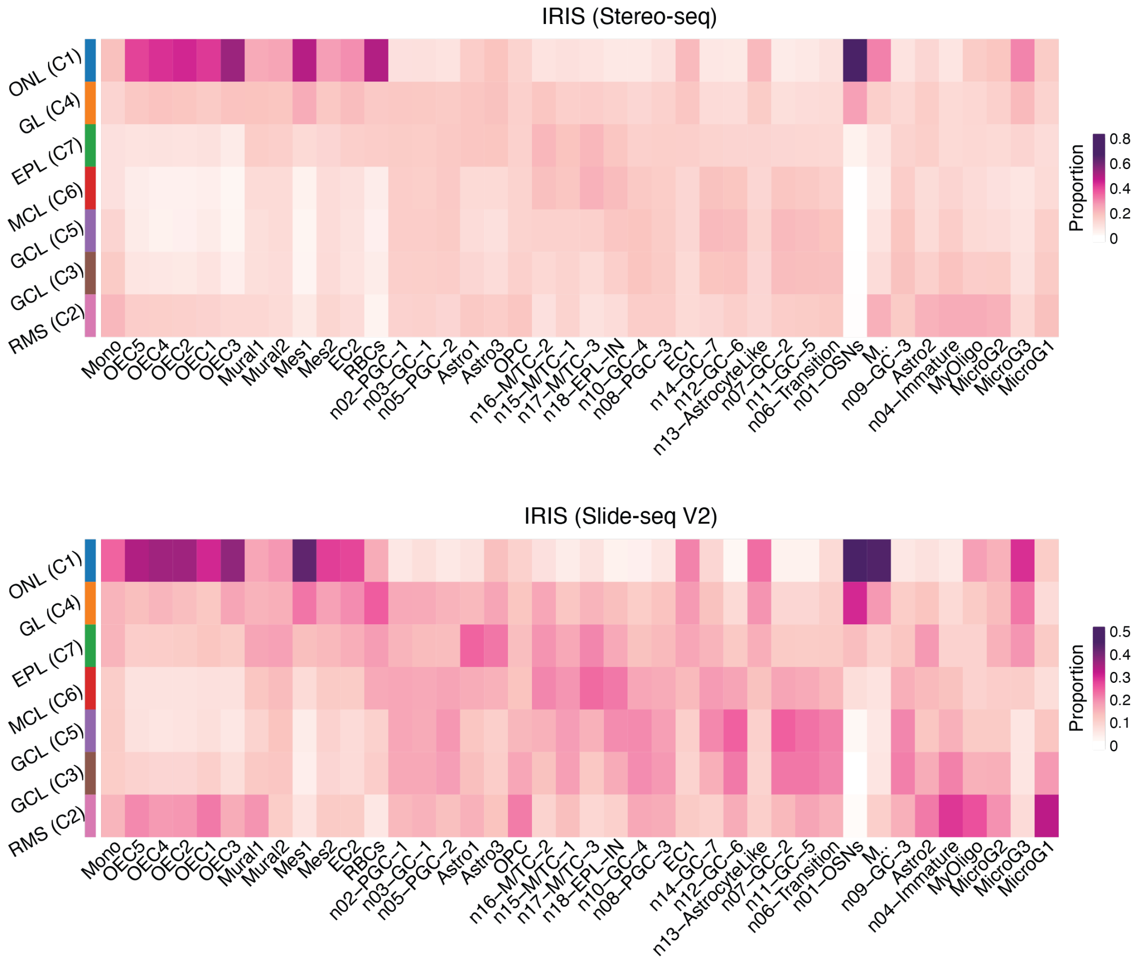

Figure S19. Heatmap plot displaying the estimated mean cell type proportion for each cell type by IRIS in each spatial domain detected by SMILE for Stereo-seq (top) and Slide-seqV2 (bottom), respectively.

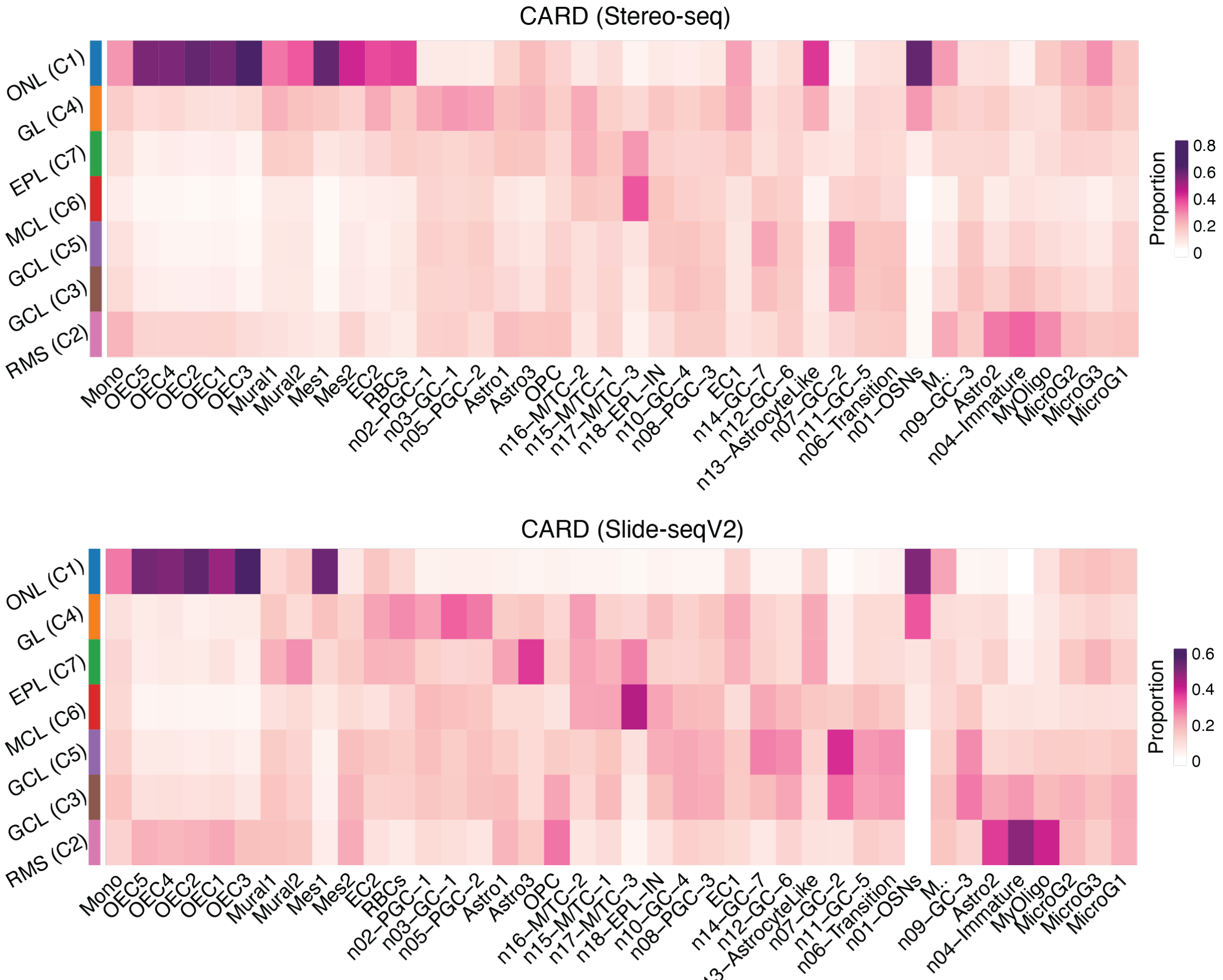

Figure S20. Heatmap plot displaying the estimated mean cell type proportion for each cell type by CARD in each spatial domain detected by SMILE for Stereo-seq (top) and Slide-seqV2 (bottom), respectively.

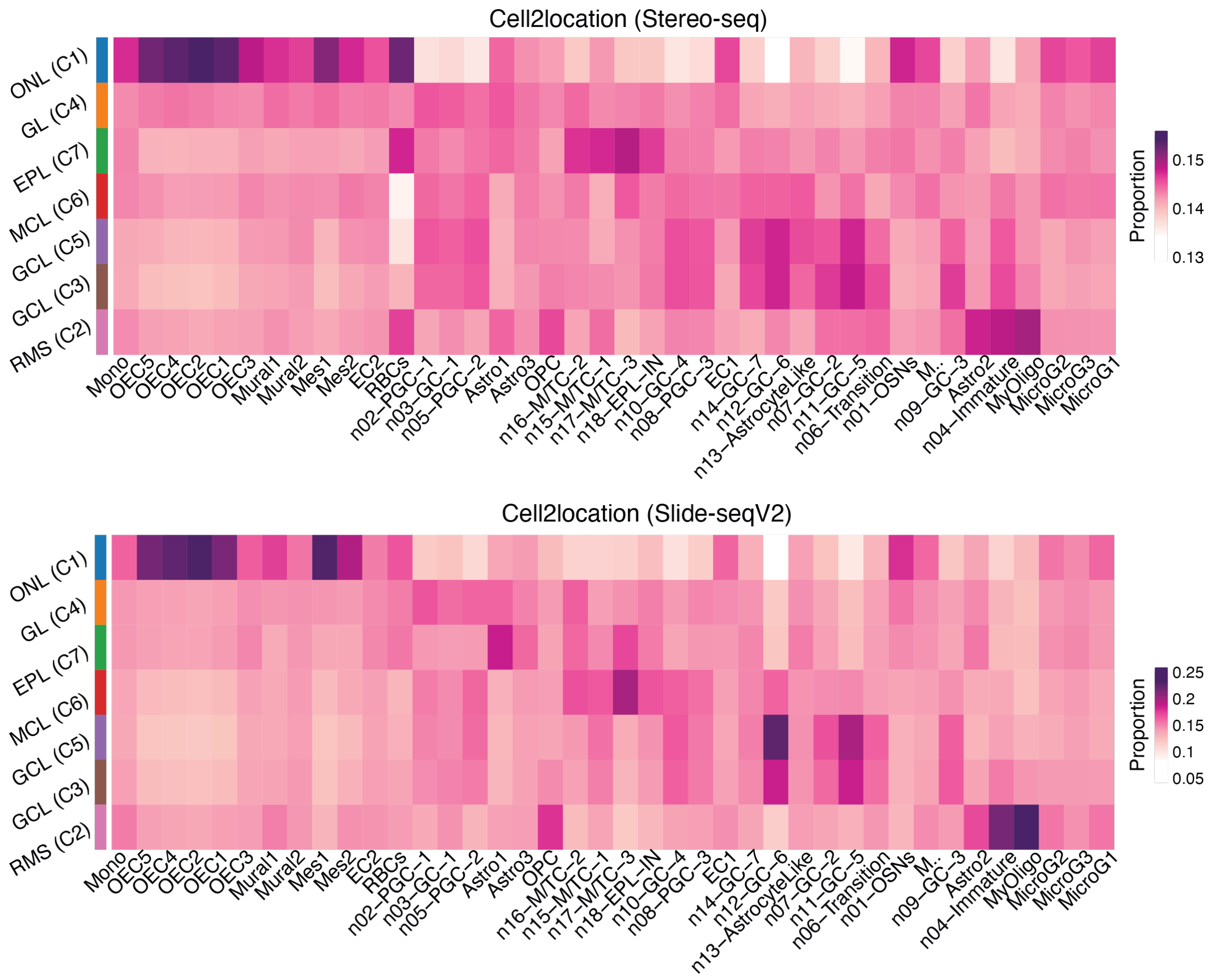

Figure S21. Heatmap plot displaying the estimated mean cell type proportion for each cell type by Cell2location in each spatial domain detected by SMILE for Stereo-seq (top) and Slide-seqV2 (bottom), respectively.

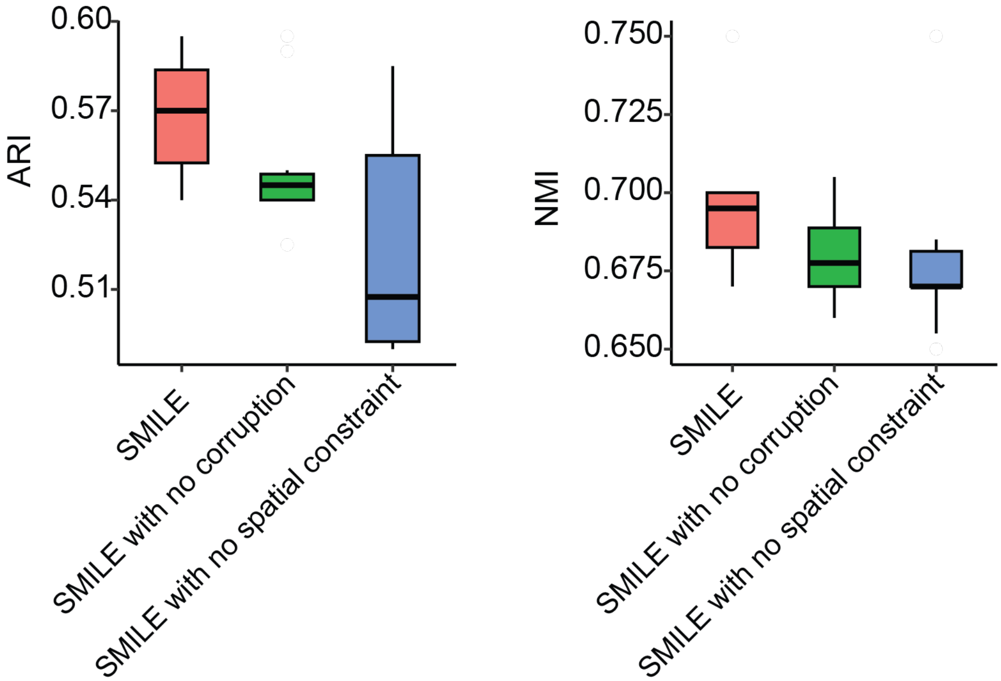

Figure S22. Ablation study on DLPFC data. We compared SMILE with two variants that do not use the corruption of the graph and spatial coordinate constraint L_R on 151508 and 151675 slices of DLPFC dataset by ARI and NMI metrics. Each experiment was implemented 10 times.

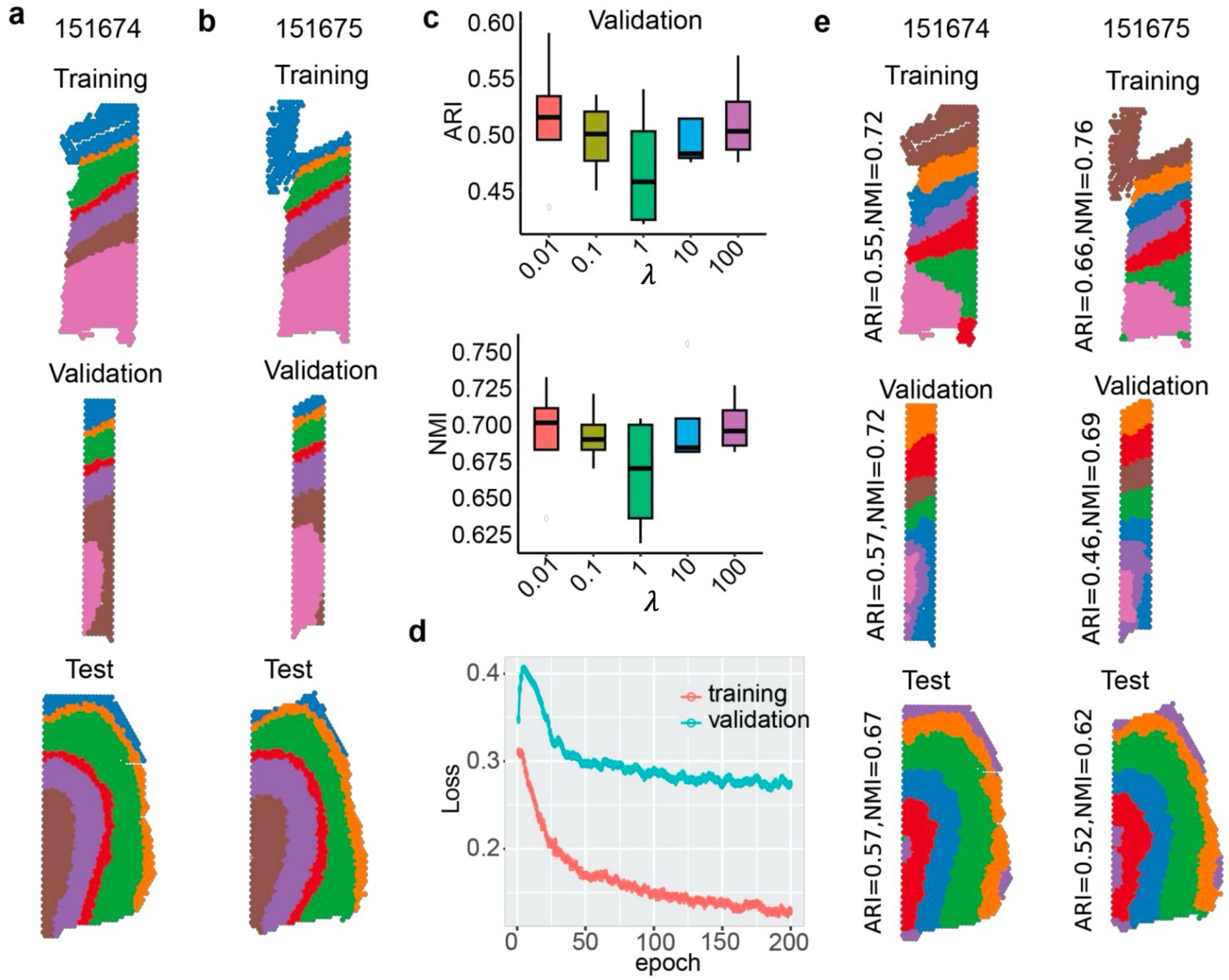

Figure S23. The performance of SMILE on training, validation and test data of DLPFC dataset. a) Spatial plot of the training, validation and test data of slice 151674, which are split based on x-axis. **b)** Spatial plot of the training, validation and test data of slice 151675. **c)** Boxplot of ARI and NMI of the pretrained SMILE on the validation data with λ varying from 0.01 to 100. We implement SMILE on training data five times with each λ value. **d)** The variation of training and validation loss with the increased epochs. **e)** The clusters identified by mclust on the embeddings of training, validation and test data sets of 151674 (left) and 151675 (right).

Supplementary Method

Simulation dataset. We generated a set of simulated spatial transcriptomic data of three slices based on real scRNA-seq data. The real scRNA-seq data was the extracted from the post-mortem brain tissue (*1*) with L2/3 IT, L4, L5 IT, L5 PT, L6b, L6CT and L6 IT. We first generated spatial location in three layers with setting the radius *r* into ranges (0,10), (10,20) and (20,30). Then generated spatial transcriptomics data with the following ratio:

$Z_{j \in S_i} = B * Y_i + \varepsilon$, *i*=1,2,3 for slice 1 and slice 2. $Z_{j \in S_3} = B * Y_4 + \varepsilon$ for slice 3, where *Y1* = *[0.25,0.25,0.5,0,0,0,0], Y2 = [0,0,0,0.5,0.5,0,0], Y3 = [0,0,0,0,0,0.5,0.5] and Y4 = [0,0,0,0,0.5,0.25,0.25].*

Parameter selection. Considering the characteristics of spatial transcriptomics data, we split each slice based on the coordinates of x-axis. We took slices 151674 and 151675 of the DLPFC dataset as an example. The data was split into training (x-axis < =45), validation (45 <x-axis<= 60) and test (x-axis >60) data sets (Supplementary Figure S23a-b). We trained SMILE on the training data and selected parameters based on the validation data. As the layer annotation of this data set was available, we used ARI and NMI values to evaluate the performance of SMILE under different parameter values. For other data without annotation information, we used the values of loss function. We took the key parameter λ as an example with its values varying from 0.01 to 100. We found that SMILE had higher ARI and NMI values with λ equaling 0.01 (Supplementary Figure S23c). Then we implemented SMILE by setting λ to 0.01 and recorded the loss on the training, validation and test data. The training and validation loss values were decreasing with the increased epochs (Supplementary Figure S23d). Next, we identified clusters by mclust on the embeddings of test data with the pre-trained SMILE. We found that SIMLE was able to detect the expected layers on the test data, which is comparable to the results on the training data (Supplementary Figure S23e).

References

1. C. Nagy *et al.*, Single-nucleus transcriptomics of the prefrontal cortex in major depressive disorder implicates oligodendrocyte precursor cells and excitatory neurons. *Nat Neurosci* **23**, 771-781 (2020).
